# ZccE is a Novel P-type ATPase That Protects *Streptococcus mutans* Against Zinc Intoxication

**DOI:** 10.1371/journal.ppat.1010477

**Published:** 2022-08-08

**Authors:** Tridib Ganguly, Alexandra M. Peterson, Marissa Burkholder, Jessica K. Kajfasz, Jacqueline Abranches, José A. Lemos

**Affiliations:** Department of Oral Biology, University of Florida College of Dentistry, Gainesville, Florida, United States of America; University of California, San Francisco, UNITED STATES

## Abstract

Zinc is a trace metal that is essential to all forms of life, but that becomes toxic at high concentrations. Because it has both antimicrobial and anti-inflammatory properties and low toxicity to mammalian cells, zinc has been used as a therapeutic agent for centuries to treat a variety of infectious and non-infectious conditions. While the usefulness of zinc-based therapies in caries prevention is controversial, zinc is incorporated into toothpaste and mouthwash formulations to prevent gingivitis and halitosis. Despite this widespread use of zinc in oral healthcare, the mechanisms that allow *Streptococcus mutans*, a keystone pathogen in dental caries and prevalent etiological agent of infective endocarditis, to overcome zinc toxicity are largely unknown. Here, we discovered that *S*. *mutans* is inherently more tolerant to high zinc stress than all other species of streptococci tested, including commensal streptococci associated with oral health. Using a transcriptome approach, we uncovered several potential strategies utilized by *S*. *mutans* to overcome zinc toxicity. Among them, we identified a previously uncharacterized P-type ATPase transporter and cognate transcriptional regulator, which we named ZccE and ZccR respectively, as responsible for the remarkable high zinc tolerance of *S*. *mutans*. In addition to zinc, we found that ZccE, which was found to be unique to *S*. *mutans* strains, mediates tolerance to at least three additional metal ions, namely cadmium, cobalt, and copper. Loss of the ability to maintain zinc homeostasis when exposed to high zinc stress severely disturbed zinc:manganese ratios, leading to heightened peroxide sensitivity that was alleviated by manganese supplementation. Finally, we showed that the ability of the Δ*zccE* strain to stably colonize the rat tooth surface after topical zinc treatment was significantly impaired, providing proof of concept that ZccE and ZccR are suitable targets for the development of antimicrobial therapies specifically tailored to kill *S*. *mutans*.

## Author summary

Dental caries is an overlooked infectious disease affecting more than 50% of the adult population. While several bacteria that reside in dental plaque have been associated with caries development and progression, *Streptococcus mutans* is deemed a keystone caries pathogen due to its capacity to modify the dental plaque environment in a way that is conducive with disease development. Zinc is an essential trace metal to life but toxic when encountered at high concentrations, to the point that it has been used as an antimicrobial for centuries. Despite the widespread use of zinc in oral healthcare products, little is known about the mechanisms utilized by oral bacteria to overcome its toxic effects. In this study, we discovered that *S*. *mutans* can tolerate exposure to much higher levels of zinc than closely related streptococcal species, including species that antagonize *S*. *mutans* and are associated with oral health. In this study, we identified a new metal transporter, named ZccE, as directly responsible for the inherently high zinc tolerance of *S*. *mutans*. Because ZccE is not present in other bacteria, our findings provide a new target for the development of a zinc-based therapy specifically tailored to kill *S*. *mutans*.

## Introduction

The first row *d*-block metal ions cobalt (Co), copper (Cu), iron (Fe), manganese (Mn), nickel (Ni) and zinc (Zn) are essential trace metals due to their roles in intracellular signaling and incorporation to structural and catalytic domains of proteins that perform the biological processes required for life [1,2]. On the flip side, these metals are toxic when present in excess, such that the ability to maintain metal homeostasis is absolutely critical for the survival of all forms of life [1–3]. To prevent or fight infections, the host activates a number of metal ion withholding strategies, collectively termed nutritional immunity, that ultimately starve pathogens of essential biometals such as Fe, Mn, and Zn [4]. For example, Fe in host fluids and tissues is sequestered by transferrin and lactoferrin, or stored within hepatocytes (reviewed in [5]). Both Mn and Zn are primarily sequestered by calprotectin, a heterodimer of the S100A8 and S100A9 proteins produced and secreted in large quantities by neutrophils (reviewed in [6]). At the same time, the host is capable of harnessing metal ions to intoxicate invading pathogens. For example, Cu and Zn are released in large quantities within phagocytic cells to kill invading pathogens [3,7].

Among the most biologically relevant trace metals, Zn is unique because it does not undergo redox-cycling. Due to its top position in the Irving-Williams series, Zn can bind more avidly and form more stable interactions with proteinaceous ligands when compared to most other metal ions. As a result, Zn is poisonous when in excess due to adventitious binding to non-cognate metalloproteins or even to residues that should not be occupied by metals, a process known as protein mismetallation [7,8]. Moreover, Zn is critical for proper functioning of the immune system as it is required for activation of pro-inflammatory signaling pathways, neutrophil extracellular trap (NET) formation and, as indicated above, it can be mobilized into phagosomes to kill invading pathogens [3,4,9]. Because it has antimicrobial properties, low toxicity to mammalian cells, and stimulates immune cell function, Zn has long been used as a therapeutic agent to treat or prevent a variety of medical conditions, from wound healing to gingivitis, or as an adjuvant in the treatment of common colds and other viral infections.

While the significance of Zn deprivation in host-pathogen interactions and the mechanisms utilized by bacteria to overcome Zn deprivation have been studied to some depth (reviewed in [8,10], the physiological consequences of Zn poisoning and the mechanisms by which pathogens overcome Zn intoxication are less well understood. In the initial response to toxic levels of Zn, bacteria have been shown to induce the expression and activity of membrane-associated Zn efflux systems to remove Zn from their cytosol. To date, three types of Zn efflux systems have been described in bacteria, namely P-type ATPases, resistance nodulation division (RND) pumps, and cation diffusion facilitator (CDF) metal/H^+^ antiporters [11]. In *Escherichia coli* (the Gram-negative paradigm), the P-type ATPase ZntA is the major Zn efflux pump, whereas *Bacillus subtilis* (the Gram-positive paradigm) has been shown to utilize both a P-type ATPase (known as CadA) and CDF antiporter (known as CzcD) to pump Zn out of the cell [12–15]. In all streptococci studied to date, a CDF-type antiporter, homologous to the *B*. *subtilis* CzcD, has been shown to serve as the major Zn-resistance determinant, with several reports showing that inactivation of *czcD* significantly diminishes bacterial virulence [9,16–22].

A resident of dental biofilms, *Streptococcus mutans* is a keystone pathogen of dental caries, one of the most prevalent and overlooked infectious diseases in the world [23,24]. In addition to its well-recognized role as a cariogenic pathogen, *S*. *mutans* is also implicated in infective endocarditis [25], with a recent retrospective study associating transient *S*. *mutans* bloodstream infections (BSI) with the highest risk of developing into infective endocarditis when compared to other streptococcal BSI [26]. In oral health, Zn has been incorporated into mouthwash and toothpaste formulations to prevent and treat halitosis and gingivitis [27,28]. In dental caries, the usefulness of Zn as a therapeutic agent is controversial, with a number of studies concluding that Zn is not effective contrasting with studies lauding Zn as a highly effective anti-plaque or anti-caries agent [29–33]. Controversy aside, mechanistic studies have shown that high concentrations of Zn inhibit the activity of glycolytic enzymes of oral streptococci *in vitro* and acid production by dental plaque *in vivo* [33–36]. Moreover, Zn and fluoride were shown to act synergistically, impairing *S*. *mutans’* acid tolerance as well as its ability to synthesize extracellular polymers of glucan from dietary sucrose [37].

Despite the widespread incorporation of Zn to oral healthcare products, very little is known about the mechanisms utilized by oral bacteria, *S*. *mutans* included, to maintain Zn homeostasis. Recently, we showed that inactivation of *adcABC*, which codes for an ABC-type Zn-specific transporter, drastically reduced the ability of *S*. *mutans* to colonize the dental biofilm in a rat model, indicating that host-mediated mechanisms for Zn sequestration, competition with microbial commensals, or both, restrict Zn availability to *S*. *mutans* in dental plaque [38]. In this report, we sought to uncover the mechanisms utilized by *S*. *mutans* to cope with toxic levels of Zn. We discovered that *S*. *mutans* exhibits much higher tolerance to Zn than all other streptococci tested, including several commensal oral streptococci associated with oral health. Through transcriptome and bioinformatic analyses, we identified a previously uncharacterized P-type ATPase transporter and cognate MerR-type transcriptional regulator as being directly responsible for the remarkable tolerance of *S*. *mutans* against Zn intoxication. *In vitro* and *in vivo* characterizations of a strain lacking this novel Zn efflux system and the fact that it is unique to *S*. *mutans* indicates that it is a potential target for the development of Zn-based antimicrobial therapies specifically tailored to kill *S*. *mutans*.

## Results

### *S*. *mutans* is inherently more tolerant to Zn intoxication when compared to other streptococci

**Fig 1:**
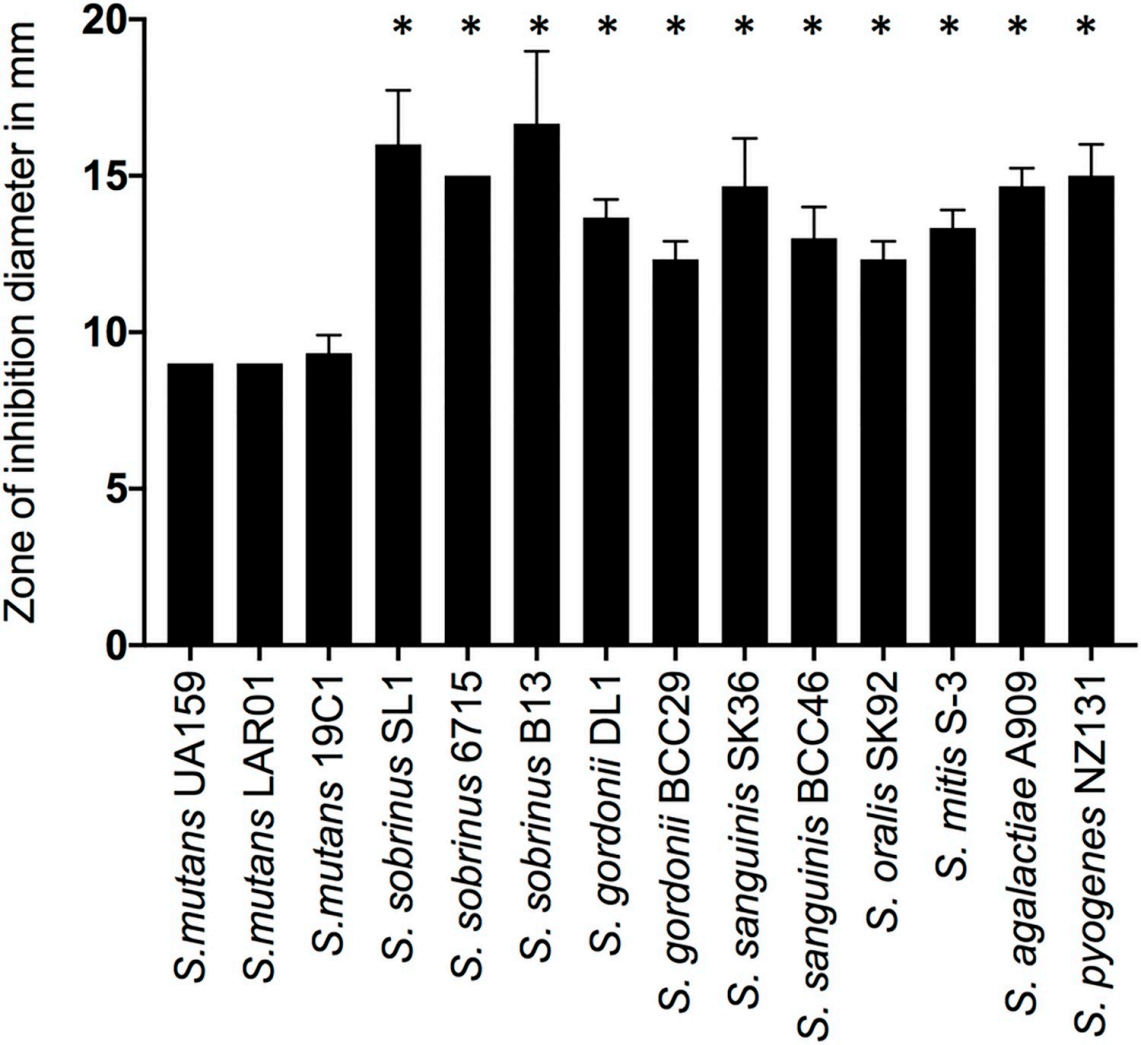
Disc diffusion assay indicating that S. mutans is inherently more tolerant to Zn than other streptococci. Cultures were grown to mid-exponential phase, spread onto BHI plates, and topped with filter paper discs saturated with 1 mM ZnSO_4_. Growth inhibition zones (in mm) around the discs were measured after 24 h incubation at 37°C in 5% CO_2_. Data represent means and standard deviations of results from 3 independent experiments. One-way ANOVA was used to compare the zones of growth inhibition of each strain to that of *S*. *mutans* UA159. A *p* value of <0.05 was considered significant (*).

To uncover the mechanisms utilized by *S*. *mutans* to cope with toxic levels of Zn, we first determined the minimum inhibitory concentration (MIC) of ZnSO_4_ in a panel of 17 streptococcal strains from 9 different species that were part of our lab collection. For the MIC determination and most other experiments, we chose BHI growth medium because it contains low levels of Zn (~10 μM) and because it supports robust growth of most streptococci. The panel included *S*. *mutans* and *S*. *sobrinus* (caries-associated streptococci), oral commensals (e.g., *S*. *gordonii*, *S*. *sanguinis*), and pyogenic streptococci *S*. *agalactiae* and *S*. *pyogenes*. Remarkably, all *S*. *mutans* strains displayed much higher Zn tolerance (MIC = 4 mM) when compared to the other species tested (MIC ranging from 0.5 to 1 mM) (**Table 1**). The intrinsically high Zn tolerance of the *S*. *mutans* strains when compared to other streptococci was further validated in disc diffusion assays and growth curve analysis (**Figs 1 and S1**).

**Table 1:** ZnSO_4_ MIC values of different streptococcal strains.

Species	Strain	ZnSO_4_ MIC
*S*. *mutans*	UA159	4 mM
*S*. *mutans*	LAR01	4 mM
*S*. *mutans*	19C1	4 mM
*S*. *mutans*	29SS	4 mM
*S*. *sobrinus*	SL1	1 mM
*S*. *sobrinus*	6715	1 mM
*S*. *sobrinus*	B13	1 mM
*S*. *gordonii*	DL1	0.5 mM
*S*. *gordonii*	K43BH3	0.5 mM
*S*. *sanguinis*	18BH1	0.5 mM
*S*. *sanguinis*	SK36	0.5 mM
*S*. *oralis*	SK92	1 mM
*S*. *mitis*	S-3	1 mM
*S*. *intermedius*	ATCC27325	0.5 mM
*S*. *agalactiae*	A909	1 mM
*S*. *pyogenes*	NZ131	0.5 mM
*S*. *mutans*	UA159Δ*zccE*	0.2 mM
*S*. *mutans*	UA159Δ*cop*	4 mM
*S*. *mutans*	UA159Δ*zccE*Δ*cop*	0.2 mM

### Overview of changes to the *S*. *mutans* UA159 transcriptome following exposure to excess Zn

**Fig 2:**
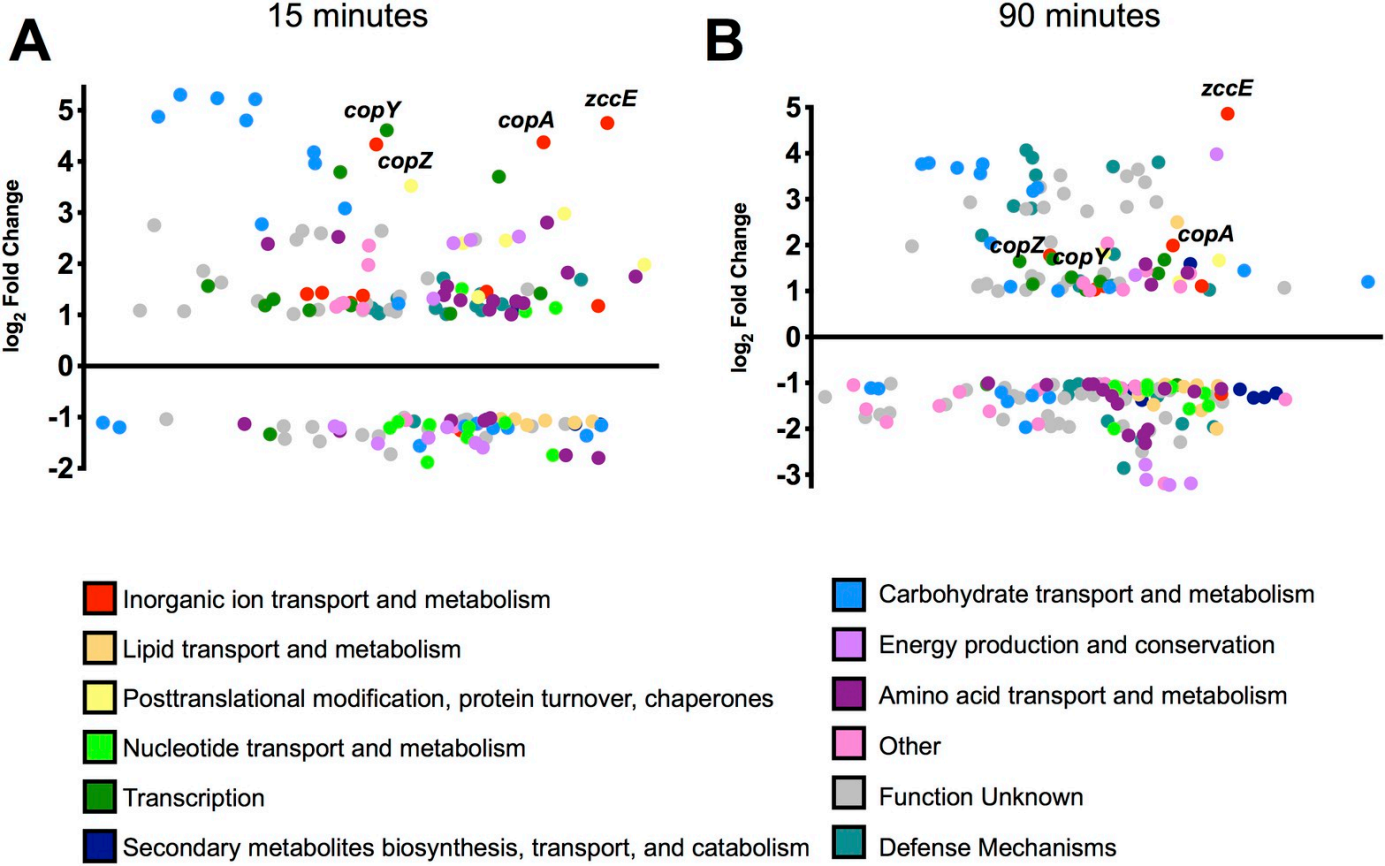
Summary of RNA-Seq analysis of *S*. *mutans* UA159 treated with 4 mM ZnSO_4_ for 15 (A) or 90 (B) minutes compared to untreated control. The y-axis indicates the log_2_ fold change in expression compared to the control, while the x-axis separates genes according to their average expression levels as compared to all other genes. The identities of selected genes of interest are indicated. The differentially expressed genes from both time points were grouped according to Clusters of Orthologous Groups (COG) functional categories [39].

To obtain mechanistic insight into the attributes that promote the high Zn tolerance of *S*. *mutans*, we used RNA deep sequencing (RNA-Seq) to uncover the transcriptome of *S*. *mutans* UA159 following exposure to excess ZnSO_4_. To determine the most appropriate concentration of Zn for this experiment, the growth kinetics of exponentially grown cultures (OD_600_ ~0.3) of *S*. *mutans* UA159 treated with increasing concentrations of ZnSO_4_ were monitored (**S2 Fig**). As exposure of exponentially grown cultures to 4 mM ZnSO_4_ resulted in a marked growth defect, but not complete growth arrest as seen at 6 mM ZnSO_4_, this concentration was chosen for the transcriptome analysis. Applying a False Discovery Rate (FDR) of 0.05 and 2-fold cutoff, we found 96 genes upregulated and 62 genes downregulated after 15 minutes (T_15_) of exposure to 4 mM ZnSO_4_ when compared to the unstressed control (**S1 Table**). After 90 minutes (T_90_), the number of differently expressed genes increased slightly from 158 at T_15_ to 199 genes, with 85 genes upregulated and 114 genes downregulated (**S1 Table**). Among the genes differently expressed, 60 genes displayed the same expression trends (up or downregulated) at both time points whereas 9 genes showed opposing trends of regulation. To illustrate these results, differentially expressed genes were grouped according to Clusters of Orthologous Groups (COG) functional categories (**Fig 2**) [39].

One of the most highly induced (greater than 25-fold upregulation) genes at both time points was *smu2057c*, coding for a putative P_1B_-type ATPase transmembrane protein. Also of interest, the entire *copYAZ* operon, of which *copA* encodes another P_1B_-type ATPase involved in Cu tolerance [40–42] were among the most upregulated genes at T_15_ (20- to 24-fold upregulated) remaining induced to a lesser extent at T_90_ (3- to 4-fold upregulated). Another group of genes highly upregulated at both time points encode for components of the lactose/galactose phosphoenolpyruvate transferase (PTS) and tagatose pathways (*lacXGEFDCBAR*, 4- to 40-fold upregulated). While genes associated with lactose uptake and utilization were strongly induced by high Zn stress, genes coding for the main PTS enzymes responsible for the uptake of sucrose (*scrA*), glucose (*manLMN*), and glucose disaccharide (*celCRB*) were downregulated at both time points. In addition, at T_15_, genes involved in the uptake or metabolism of cysteine (*tycABC*, *tcyEFGH*, *cysK*) and glutathione (*gst*, *gshT*, *gor*) were upregulated by 2- to 5-fold whereas genes encoding for mutacins IV (*nlmD*), V (*nlmC*) and VI (*nlmAB*) were induced by 8- to 16-fold at T_90_. Finally, several stress genes were induced at T_15_, including genes coding for heat shock proteins/molecular chaperone (*groES*-*EL* and *hrcA*-*grpE*-*dnaK*-*dnaJ* operons) and oxidative stress genes (*dpr*, *gor* and *tpx*). In contrast, the same *dpr* and *tpx* genes induced at T_15_ and additional genes classically associated with oxidative stress responses (*ahpCF*, *nox* and *sodA*) were downregulated at T_90_.

### *smu2057c* encodes a multi-metal translocating P-type ATPase unique to *S*. *mutans*

**Fig 3:**
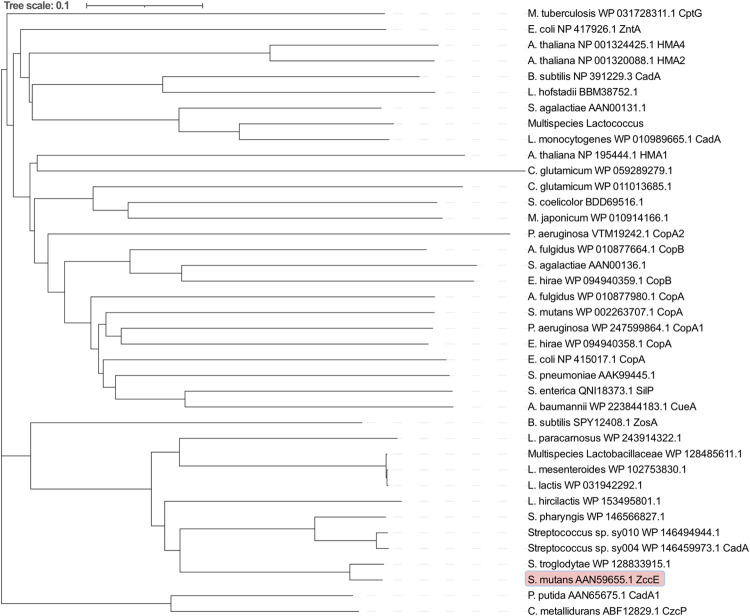
Phylogenetic tree of P-type ATPases including ZccE and 38 other P-type ATPases identified from the literature and from BLASTP searches for comparison. The tree distance is the calculated distance from the multiple sequence alignment in Clustal Omega. ZccE is highlighted in red.

As *smu2057c* was one of the most strongly upregulated genes during high Zn stress and knowing that a handful of bacterial P_1B_ -type ATPases are implicated in metal ion transport [13–15,43], we predicted that Smu2057c would function as a Zn efflux system. Based on searches of public databases, Smu2057c is exclusively found in *S*. *mutans* genomes, with the exception of a single *Streptococcus troglodytae* genome encoding a hypothetical protein that was 94% identical to Smu2057c. A phylogenetic analysis confirmed that ZccE is evolutionarily distant from other P-type ATPases available on public databases (**Fig 3**). Compared to P_1B_-type ATPases previously implicated in metal tolerance, the protein coded by *smu2057c* shares 33% amino acid identity with the *B*. *subtilis* CadA, which mediates tolerance to cadmium (Cd), cobalt (Co) and Zn [14,44], and 36% identity to *E*. *coli* ZntA that mediates tolerance to Cd, lead (Pb) and Zn [13,15] (**S3 Fig**). Finally, we determined the predicted structure of ZccE using AlphaFold2 and compared it to the available structure of ZntA (**S4 Fig**). Despite the similarities at the amino acid level, the two structures are distinctly different.

**Fig 4:**
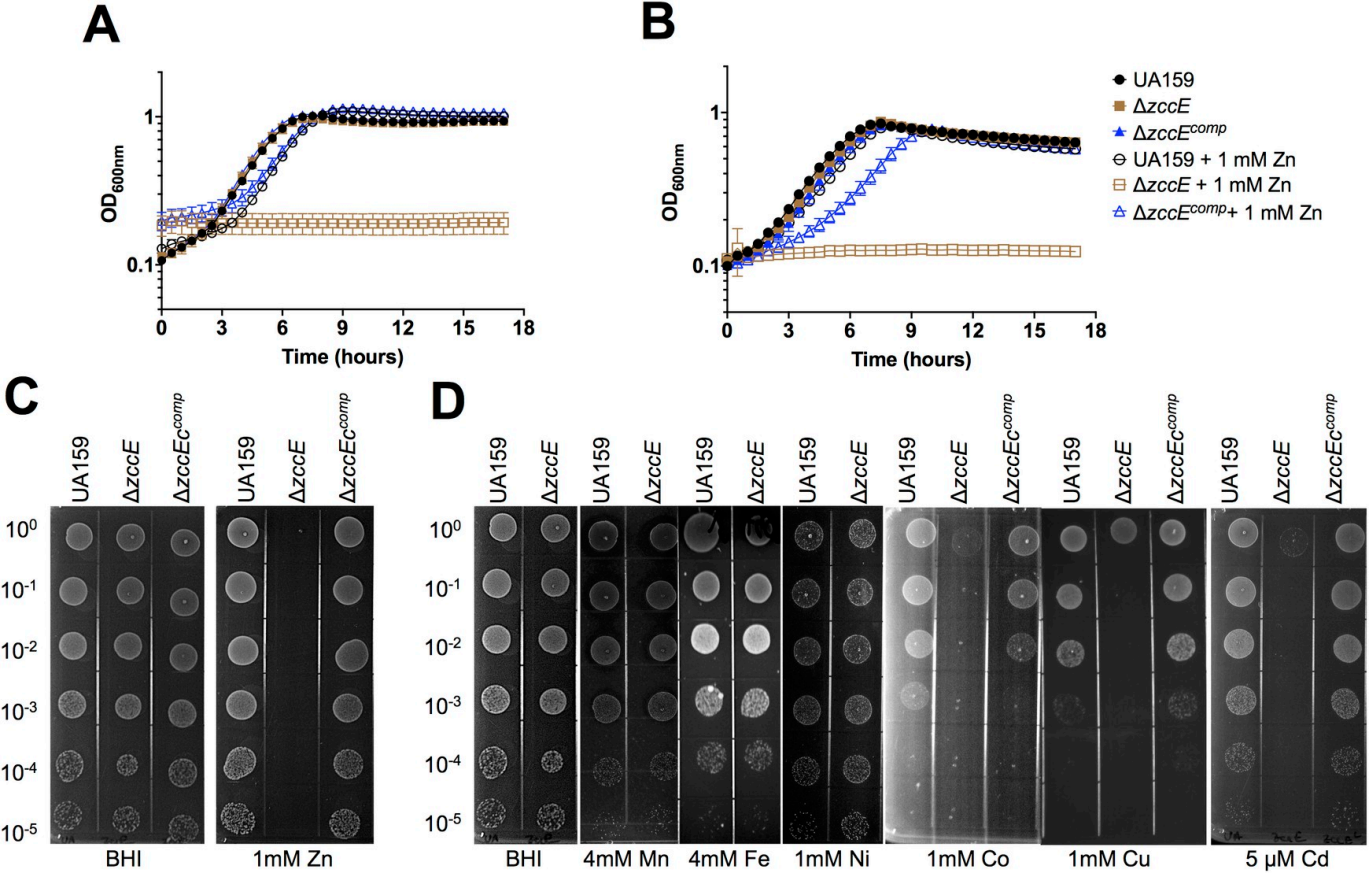
ZccE is a multi-metal exporter responsible for the high Zn tolerance of *S*. *mutans*. (A-B) Growth curves of *S*. *mutans* UA159, Δ*zccE* and Δ*zccE*^comp^ strains in FMC (A) or BHI (B) with or without 1 mM Zn supplementation. Data represent means from 3 independent experiments and the error bars represent standard deviations of the results. A nonlinear curve fit analysis and measuring the doubling time indicated that the growth defect of the Δ*zccE* strain was statistically significant when compared to either UA159 or Δ*zccE*^comp^ strains. (C) Plate titration (spot test) of *S*. *mutans* UA159, Δ*zccE* and Δ*zccE*^comp^ strains on BHI agar with or without 1 mM Zn supplementation. (D) Plate titration of *S*. *mutans* UA159 and Δ*zccE* strains on BHI agar supplemented with 4 mM Mn, 4 mM Fe, 1 mM Cu, 1 mM Ni or 5 μM Cd. (C-D) Images were taken after 24 h incubation at 37°C in 5% CO_2_ and are representative of at least 3 independent experiments.

To determine if Smu2057c mediates Zn tolerance, we generated a *smu2057* deletion strain in the UA159 wild-type background and then tested the capacity of the mutant to grow in chemically-defined (FMC) or complex (BHI) media containing increasing concentrations of Zn. Because the experiments described below implicate Smu2057c in Cd, Co, Cu and Zn tolerance, we named *smu2057c* as *zccE*, for zinc, cadmium, cobalt, and copper exporter. When compared to UA159, the Δ*zccE* strain displayed a much higher sensitivity to Zn as it was completely unable to grow in media supplemented with 1 mM ZnSO_4_ (**Fig 4A–4C**). In agreement with the growth curves and plate titration assay, the Zn MIC of Δ*zccE* was 20 times lower than the MIC of the parent strain (**Table 1**). Genetic complementation of Δ*zccE* (Δ*zccE*^Comp^ strain) fully rescued the Zn sensitive phenotype (**Fig 4A–4C**), while inactivation of *zccE* in the *S*. *mutans* OMZ175 background strain led to comparable and drastic reduction in Zn tolerance supporting that ZccE is the primary Zn tolerance determinant of *S*. *mutans* (**S5 Fig**). Because P-type ATPases implicated in Zn tolerance have been shown to confer resistance to other metal ions, especially those of similar size and charge, we also tested the capacity of Δ*zccE* to grow on BHI plates supplemented with Cd, Co, Cu, Fe, Mn, and Ni. As shown in **Fig 4D**, ZccE confers resistance to Cd, Co, and Cu but not to Fe, Mn, or Ni. The increased sensitivity of Δ*zccE* to Cd, Co and Cu was restored in the complemented strain.

### ZccE and CopA work synergistically to protect *S*. *mutans* against Cu intoxication in oxidizing environments

**Fig 5:**
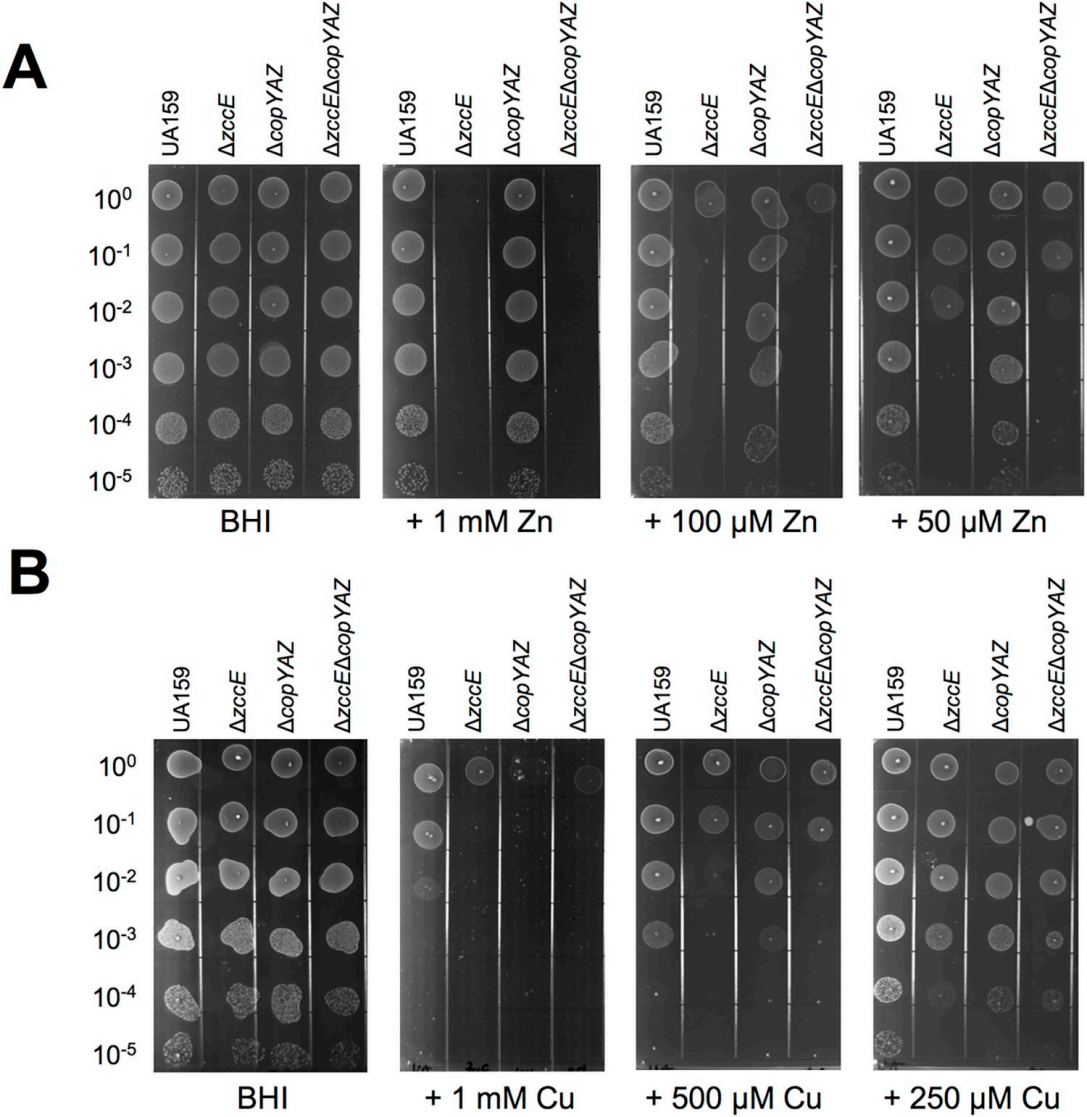
CopA plays a minor role in Zn tolerance while ZccE and CopA work interchangeably to protect against Cu intoxication in oxidizing environments. (A-B) Plate titration (spot test) of *S*. *mutans* UA159, Δ*zccE*, Δ*copYAZ*, and Δ*zccE*Δ*copYAZ* strains on BHI agar with varying concentrations of Zn (A) or Cu (B). Images were taken after 24 h incubation at 37°C in 5% CO_2_ and are representative of at least 3 independent experiments.

In several bacteria, including all streptococci, Cu tolerance is mediated by the Cu-translocating P-type ATPase encoded by the *copA* gene, which is organized in an operon with *copY* (Cu-sensing transcriptional repressor) and *copZ* (Cu chaperone) [45–47]. Because we found that ZccE also confers Cu tolerance and that the *copYAZ* operon was induced upon high Zn stress, we sought to determine to what extent ZccE contributes to Cu tolerance and to probe the possible association of CopA with Zn tolerance. To this end, we deleted the entire *copYAZ* operon in both the UA159 and Δ*zccE* background strains to generate the Δ*copYAZ* and Δ*zccE*Δ*copYAZ* strains. Next, we tested the capacity of each mutant strain to grow in media containing high concentrations of CuSO_4_ or ZnSO_4_. Despite strong upregulation of the *copYAZ* operon during the initial 15 minutes of high Zn stress (**S1 Table, Fig 2**), inactivation of *copYAZ* alone did not affect Zn sensitivity (**Fig 5A**). While the Δ*zccE*Δ*copYAZ* strain phenocopied Δ*zccE* on plates containing 0.1 or 1 mM ZnSO_4_, the double mutant was slightly more sensitive than the Δ*zccE* single mutant on plates containing 0.05 mM ZnSO_4_ (**Fig 5A**). Collectively, these results indicate that CopA plays a minor role in Zn tolerance that becomes dispensable when ZccE is present.

As shown by others [40,45], inactivation of the *copYAZ* operon drastically heightened Cu sensitivity albeit the Δ*zccE* strain was slightly more sensitive than Δ*copYAZ* on plates containing 500 μM CuSO_4_ (**Fig 5B**). Unexpectedly, inactivation of both *copYAZ* and *zccE* did not appear to further enhance Cu sensitivity when compared to the single mutant strains (**Fig 5B**). Though Cu should primarily be found as Cu^+^ in the reducing bacterial cytoplasm [48], it is capable of alternating between Cu^+^ or Cu^2+^. Thus, we wondered if ZccE could mobilize both oxidized and reduced Cu and, if so, whether there was a preference for one redox state over another. Of note, previous work has shown that the pneumococcal Cop operon can efficiently remove Cu^+^ and Cu^2+^ from the bacterial cytoplasm into the extracellular milieu [49]. In light of this, we reevaluated the Cu tolerance of Δ*zccE* and Δ*copYAZ* single and double mutants under anaerobiosis such that all Cu ions available should be in the reduced Cu^+^ form. In agreement with the knowledge that Cu^+^ is more toxic than Cu^2+^, supplementation of agar plates with 1 mM CuSO_4_ caused complete growth inhibition of all strains, such that experiments using an anaerobic environment were conducted with plates containing at most 0.1 mM CuSO_4_. Interestingly, we found that Cu tolerance under anaerobiosis was solely mediated by CopA, suggesting that ZccE cannot efficiently mobilize Cu^+^ (**S6 Fig**). Taken together, these results reveal that CopA and ZccE interchangeably mediate Cu protection in aerobic environments. However, CopA is likely to have greater biological relevance to Cu tolerance than ZccE due to its capacity to export both Cu^+^ and Cu^2+^.

### ZccE is positively regulated by a MerR-type regulator

**Fig 6:**
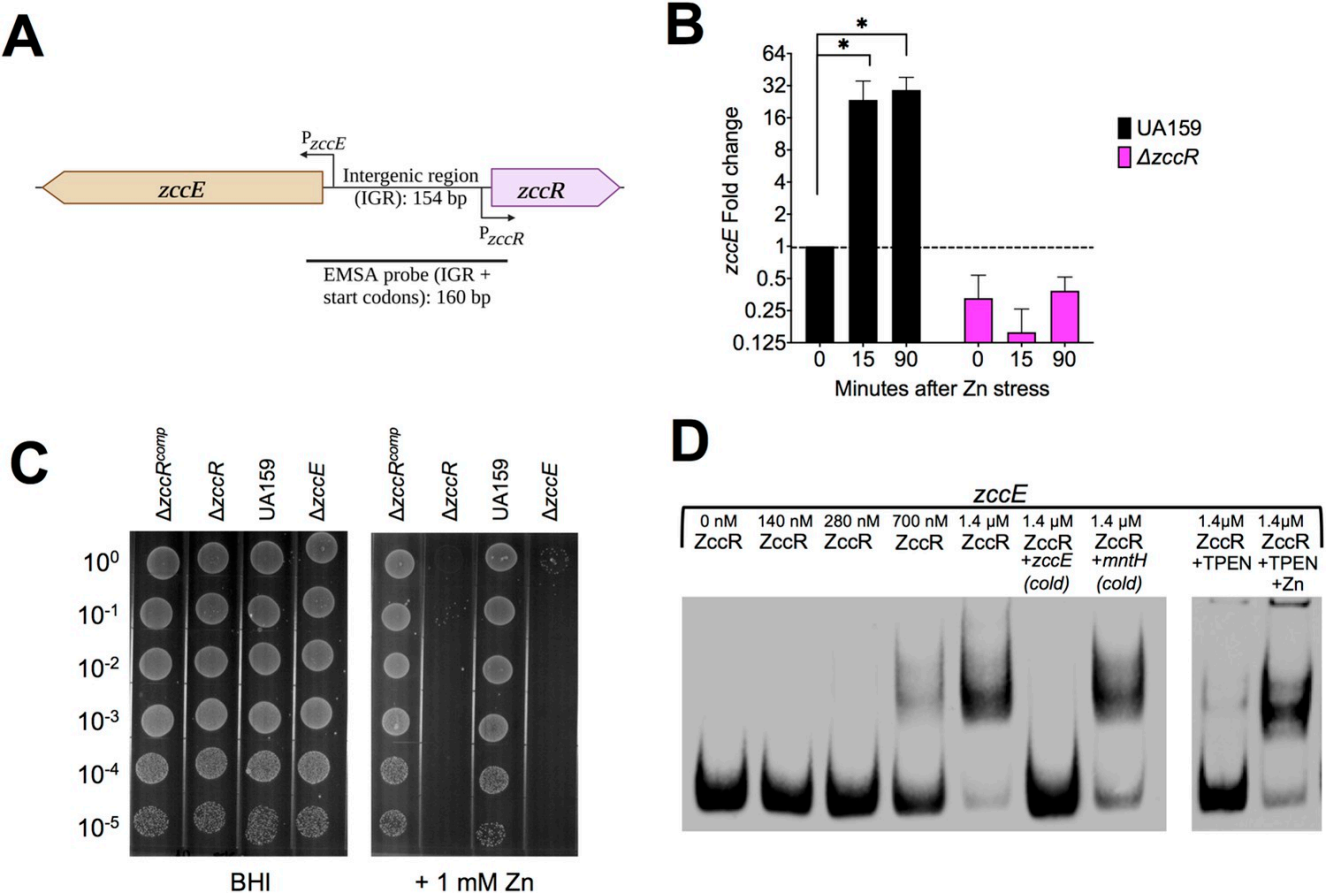
The MerR-type transcription factor ZccR is a positive regulator of *zccE*. (A) Genetic organization of *zccE* (*smu2057c*) and *zccR* (*smu2058*) in the *S*. *mutans* UA159 chromosome (Created with BioRender.com). (B) qRT-PCR analysis of *zccE* mRNA expression in the Δ*zccR* strain relative to the parent UA159 strain before and after exposure to 4 mM Zn. Data represent means and standard deviations of results from 3 independent experiments. One-way ANOVA was used to determine significance (*, *p* <0.001; ns, not significant). (C) Plate titration (spot test) of UA159, Δ*zccE*, Δ*zccR* and Δ*zccR*^*comp*^ strains on BHI with or without 1 mM Zn supplementation. Images were taken after 24 h incubation at 37°C in 5% CO_2_ and are representative of at least 3 independent experiments. (D) EMSA showing direct interaction of ZccR with the 160-bp *zccE*-*zccR* intergenic region (IGR). To determine ZccR binding specificity, addition of 100X (molar excess) of either non-biotin labeled (cold) *zccE*-*zccR* IGR or non-specific competitor DNA (*mntH* promoter region) was added to the reaction. To explore the role of Zn on ZccR binding activity, 3 mM TPEN (Zn specific chelator) was added to the reaction alone or in the presence of 2.5 mM ZnSO_4_.

Sequence analysis revealed that *zccE* is located immediately upstream but transcribed in the opposite orientation of *smu2058*, an uncharacterized MerR-type transcriptional regulator (**Fig 6A**). To explore a possible role of Smu2058 in *zccE* regulation, the *smu2058* coding sequence was replaced by a Spec^R^ cassette generating the Δ*zccR* strain (for *zcc**E* regulator). Using the same growth conditions as were used in the RNA-Seq analysis described above, quantitative real time PCR (qRT-PCR) analysis revealed that *zccE* transcription is almost entirely dependent on ZccR (**Fig 6B**). In agreement with this observation, the Δ*zccR* strain phenocopied the Δ*zccE* strain and was unable to grow in broth (**S7 Fig**) or on agar plates (**Fig 6C**) supplemented with Zn. Genetic complementation of Δ*zccR* (Δ*zccR*^Comp^ strain) reversed the heightened Zn sensitivity indicating that the phenotype was not due to polar effects on *zccE* or secondary mutations (**Figs S7 and** **6C**).

To determine if ZccR regulation of *zccE* transcription is direct or indirect, we performed electron mobility shift assays (EMSAs) using recombinant ZccR purified from *E*. *coli* and a DNA probe encompassing the entire *zccE*-*zccR* intergenic region (**Fig 6A**). The EMSA results revealed that addition of sufficient ZccR resulted in a shift of the protein: DNA complex, indicating that ZccR binds specifically to the *zccE*-*zccR* intergenic region (**Fig 6D**). Formation of the ZccR:DNA complex was inhibited by the Zn-specific chelator TPEN (*N*,*N*,*N′*,*N′*-tetrakis(2-pyridinylmethyl)-1,2-ethanediamine) and competitively reverted by the excess Zn supplementation (**Fig 6D**). Thus, we conclude that ZccR positively and directly regulates *zccE* expression, likely by sensing and responding to intracellular Zn pools.

### Inactivation of *zccE* or *zccR* drastically alters Zn:Mn ratios in high Zn conditions

**Fig 7:**
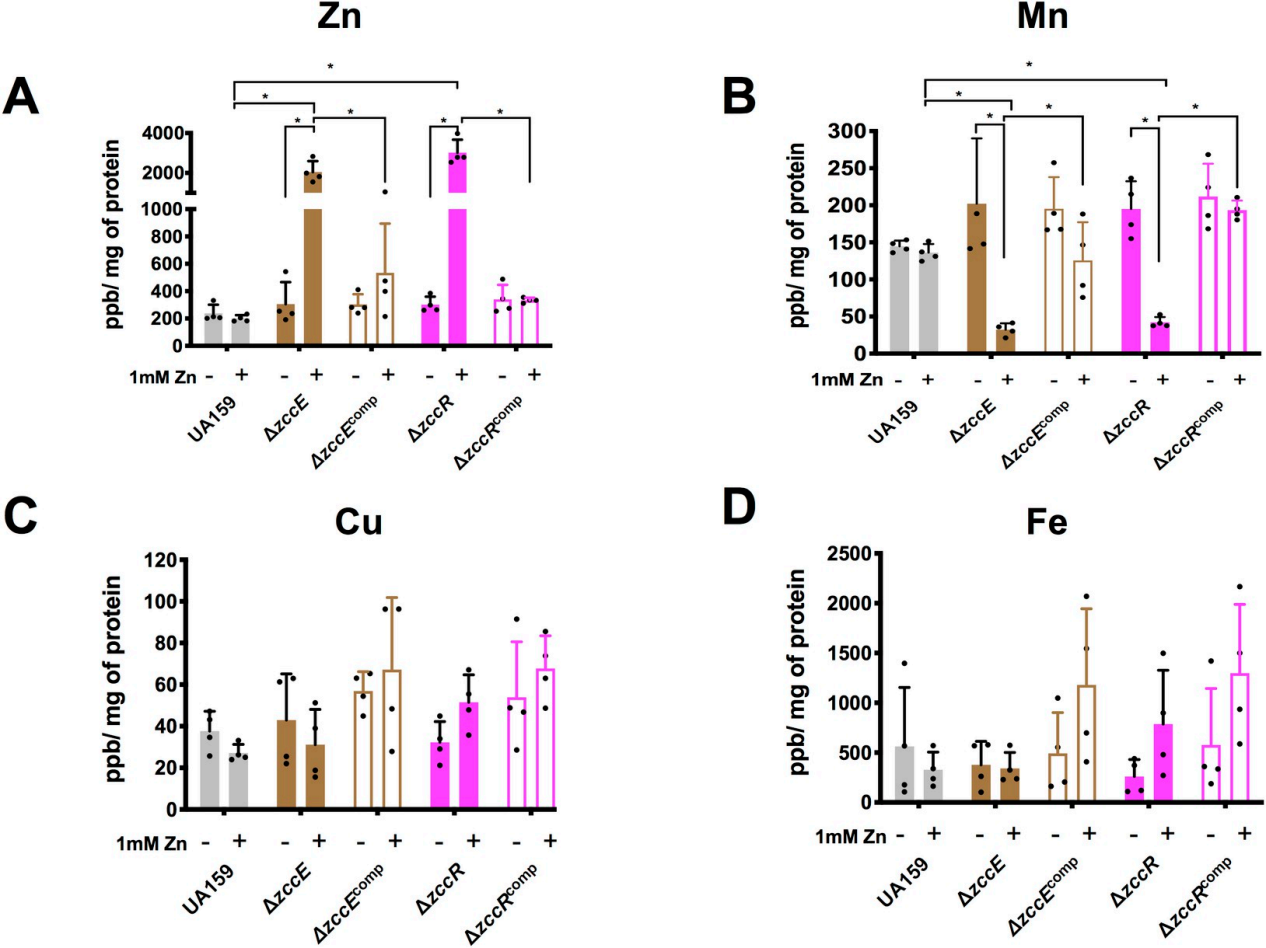
ICP-MS quantifications of intracellular Zn, Mn, Cu and Fe in UA159, Δ*zccE*, Δ*zccR* and respective complemented strains. Strains were grown in BHI to mid-exponential phase at which point the experimental groups were exposed to 1 mM Zn for 90 minutes. The graphs indicate intracellular parts per billion Zn (A), Mn (B), Cu (C), and Fe (D) normalized by milligram of total protein. Data represent average and standard deviation of values from four independent biological replicates. Two-way ANOVA was used to determine significance between metal content of either the same strain before and after Zn exposure or among different strains after Zn exposure. Error bars represent standard deviations of results from at least 3 independent experiments. A *p* value of <0.05 was considered significant (*), and only comparisons that were statistically significant are indicated in the figure.

**Fig 8:**
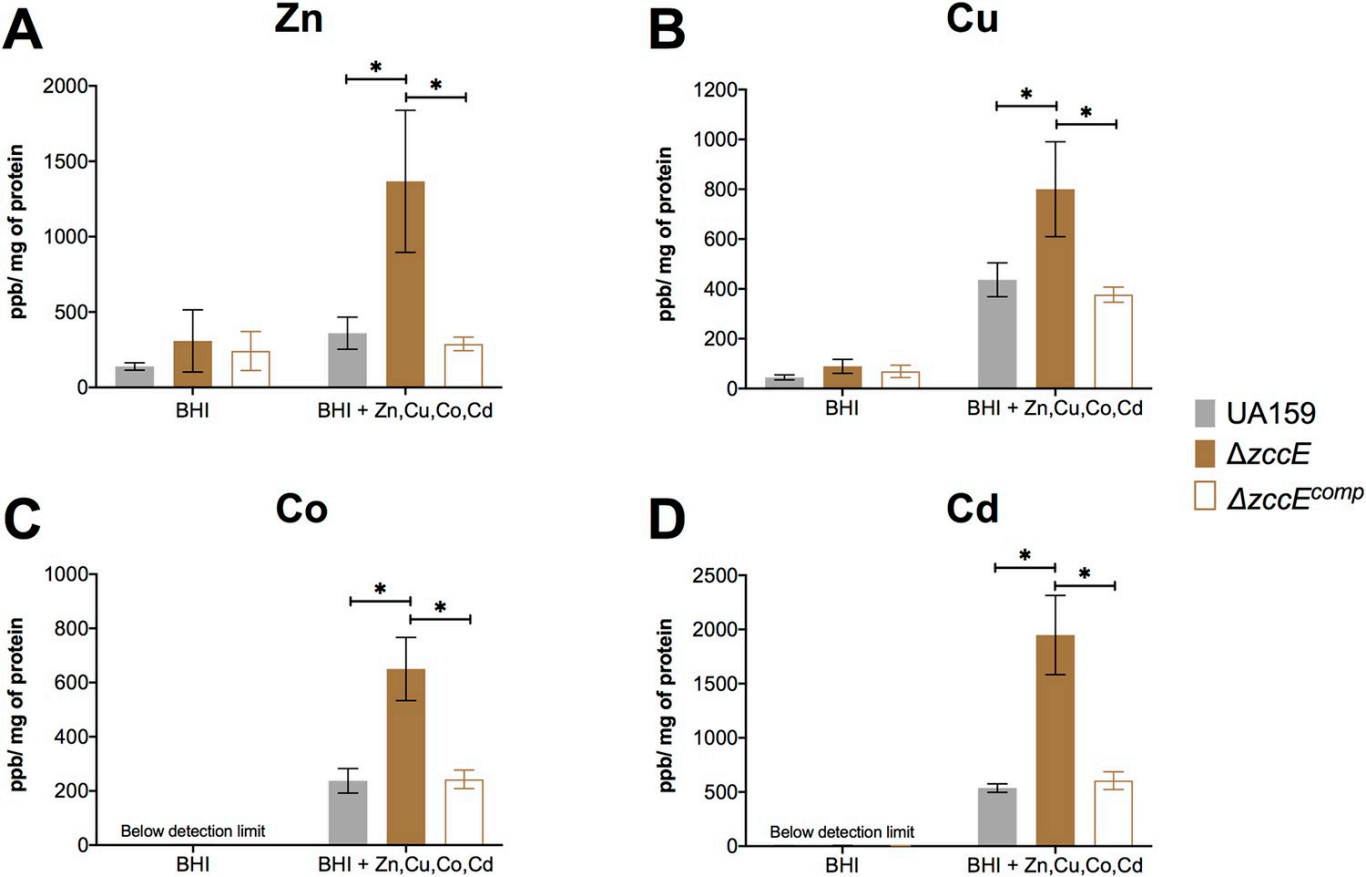
ICP-MS quantifications of intracellular Zn, Cu, Co, and Cd in UA159, Δ*zccE*, and complemented strains. Strains were grown in BHI to mid-exponential phase at which point the experimental groups were exposed to a mixture of sub-inhibitory concentrations of the 4 metals (0.5 mM Zn, 0.5 mM Cu, 0.5 mM Co and 2.5 μM Cd) for 90 minutes. The graphs indicate intracellular parts per billion of Zn (A), Cu (B), Co (C) and Cd (D) normalized by milligram of total protein. Two-way ANOVA was used to determine significant differences in intracellular metal content among strains after metal exposure. A *p* value of <0.05 was considered significant (*). Average and error bars represent standard deviations of results from at least 3 independent experiments.

Next, we used inductively coupled plasma mass spectrometry (ICP-MS) to quantify the intracellular trace metal pools in the UA159 (parent strain), Δ*zccE* and Δ*zccR* strains exposed to high Zn stress. Briefly, cultures were grown in BHI broth (~ 10 μM Zn,~ 0.5 μM Mn [50]) to mid-log phase and treated with a ZnSO_4_ solution to reach a final concentration of 1 mM of Zn. For ICP-MS analysis, samples were obtained immediately before (control) and 90 minutes after addition of Zn. Despite the high concentration of Zn added to the culture, parent and complemented mutant strains maintained the same intracellular amounts of Zn before and after the Zn challenge (**Fig 7A**). While intracellular levels of Zn in the Δ*zccE* and Δ*zccR* strains before addition of Zn did not differ from parent and complemented mutants, intracellular Zn pools rose by ~10-fold in the mutants after Zn challenge (**Fig 7A**). Moreover, accumulation of Zn in the Δ*zccE* and Δ*zccR* strains inversely correlated with a substantial decrease (~10-fold) in intracellular Mn pools (**Fig 7B**). The intracellular levels of Cu or Fe did not alter upon Zn stress either in the parent strain UA159 and Δ*zccE* (**Fig 7C & 7D**), while other d-block elements such as Co and Cd were below the detection limit. To determine whether the other 3 metals (Cd, Co, and Cu) accumulate in Δ*zccE* when provided in excess, we expanded our ICP-MS analysis to include cells grown in BHI supplemented with a combination of the 4 metals (Zn, Co, Cu, and Cd) associated with ZccE activity. As shown in **Fig 8,** the Δ*zccE* strain accumulated significantly more Cd, Co, Cu and Zn compared to parent or complemented strains.

### Disruption of Zn homeostasis diminishes oxidative stress tolerance

**Fig 9:**
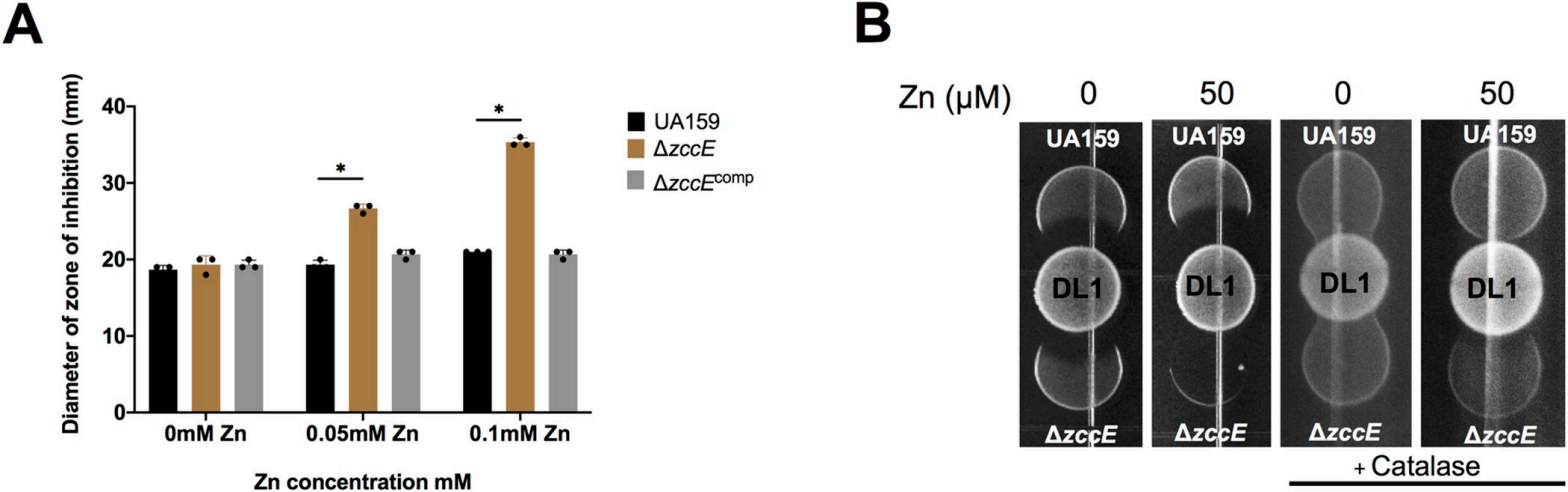
Disruption of Zn homeostasis increases H_2_O_2_ sensitivity in the *zccE* strain. (A) Growth inhibition zones for *S*. *mutans* UA159, Δ*zccE*, and Δ*zccE*^Comp^ strains grown on BHI agar with or without Zn supplementation and exposed to filter paper discs saturated with 0.25% H_2_O_2_. Two-way ANOVA was used to determine significance (*, *p* <0.05). (B) Growth inhibition zones of *S*. *mutans* UA159 and Δ*zccE* strains by peroxigenic *S*. *gordonii* DL-1 spotted on BHI agar with or without 50 μM Zn supplementation. The *S*. *mutans*-*S*. *gordonii* competition assay was repeated with catalase overlaid onto the *S*. *gordonii* spot to inactivate H_2_O_2_. Images are representative of results from three independent experiments.

As both Zn and Mn are linked to oxidative stress responses [50–52], we sought to determine if simultaneous perturbations of Zn and Mn homeostasis (and therefore of Zn:Mn ratios) in Δ*zccE* affected the ability of this strain to cope with peroxide stress. In disc diffusion assays, both UA159 and Δ*zccE* strains displayed similar sensitivity to H_2_O_2_ when grown in BHI alone (~ 10 μM Zn) while supplementation of sub-inhibitory concentrations of Zn (50 to 100 μM ZnSO_4_) resulted in significantly greater sensitivity of Δ*zccE* when exposed to H_2_O_2_ (**Fig 9A**). Next, we tested the ability of *S*. *gordonii* DL1, a peroxigenic oral commensal, to inhibit the growth of the *S*. *mutans* parent and Δ*zccE* strains using an antagonism plate assay. Like the disc diffusion assay, both strains showed similar sensitivities to *S*. *gordonii* when grown on BHI plates, while supplementation of the media with as little as 50 μM ZnSO_4_ virtually abolished growth of Δ*zccE* (**Fig 9B**). To confirm that the growth defect was due to sensitivity to the H_2_O_2_ produced by *S*. *gordonii*, a control assay was performed in which catalase was added atop the *S*. *gordonii* prior to spotting of the *S*. *mutans* cultures. When the H_2_O_2_ was neutralized by catalase, no growth sensitivity by the *S*. *mutans* strains was observed (**Fig 9B**). To quantify the effect of Zn in *S*. *gordonii* competition we also measured CFUs after 24 hours of incubation of *S*. *mutans* UA159, Δ*zccE* and Δ*zccE*^*comp*^ in BHI containing increasing concentrations of Zn (0, 50 and 100 μM Zn) either in monoculture or co-culture with *S*. *gordonii* (1:2 ratio of *S*. *mutans*:*S*. *gordonii*). As expected, only the Δ*zccE* strain became more susceptible to *S*. *gordonii* killing after the addition of excess Zn (**S8 Fig**).

### Topical Zn treatment impairs oral colonization efficiency of the *ΔzccE* strain

**Fig 10:**
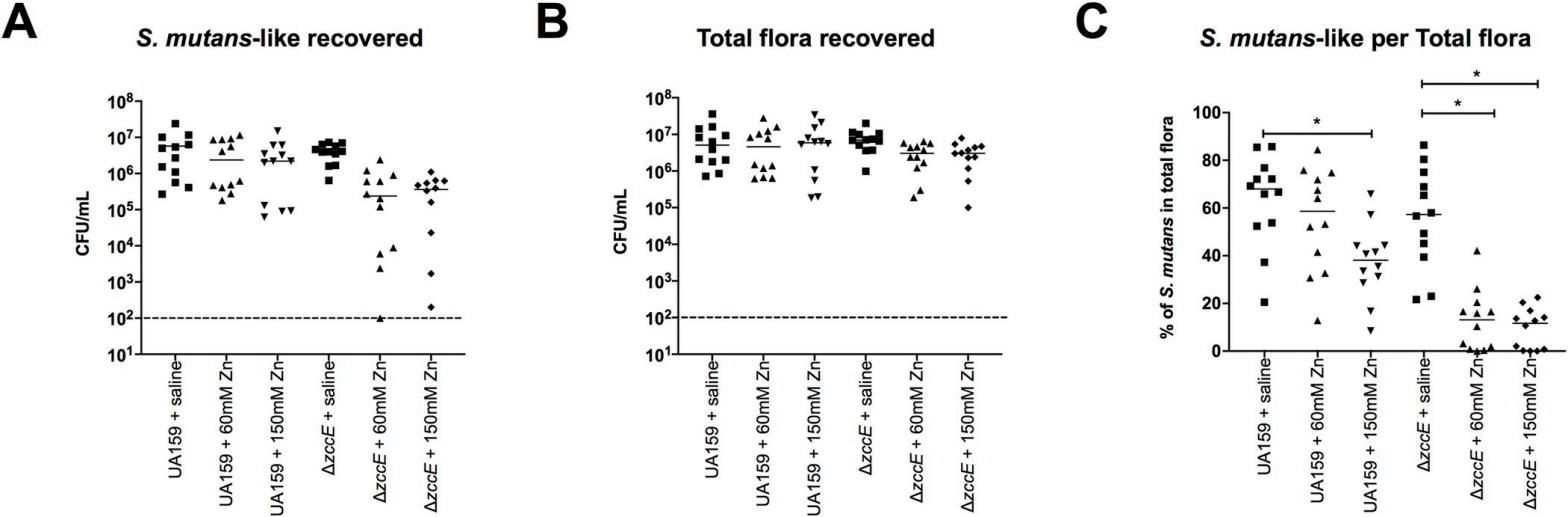
Colonization efficiency of *S*. *mutans* UA159 or Δ*zccE* on the teeth of rats after topical treatment with Zn. (A-B) Bacterial CFU recovered from rat jaws by plating on (A) MS agar for *S*. *mutans* and (B) blood agar for total flora. (C) Percentage of *S*. *mutans*-like colonies over total flora were calculated by dividing the values shown in (A) by those shown in (B). The dashed line indicates the limit of detection. One-way ANOVA was used to determine significance, and only statistically significant comparisons with *p* value <0.05 (*) are indicated in the figure.

To determine the impact of Zn homeostasis in an *in-vivo* model, we assessed the ability of the parent and Δ*zccE* strains to colonize the oral cavity of rats fed a cariogenic diet, while simultaneously testing whether topical Zn treatment could inhibit bacterial colonization (**Fig 10**). To simulate oral hygiene such as a mouthwash, rats that had been orally infected with *S*. *mutans* UA159 or *ΔzccE* were treated twice daily with an oral application of a ZnSO_4_ solution. When comparing the control treatment groups (saline), similar numbers of colony-forming units (CFU) were recovered from animals infected with either UA159 or Δ*zccE*, indicating that the multi-metal tolerance conferred by ZccE was dispensable for oral colonization in the absence of excess Zn. However, daily treatment with 60 mM or 150 mM ZnSO_4_ solutions, a concentration range found in commercially available oral healthcare products, resulted in significant reduction (~1-log) of the CFU recovered from rats that had been infected with the Δ*zccE* strain. These same Zn treatments showed only modest, and not statistically significant, inhibitory effects against the parent strain. Importantly, Zn treatment did not affect the abundance of total flora recovered from the *S*. *mutans*-infected animals, suggesting that the inhibitory effect was specific to the Δ*zccE* strain. Taken together, these results provide the proof of concept that ZccE is a suitable target for future development of an antimicrobial therapy specific to treat *S*. *mutans* infections.

## Discussion

The second most abundant trace metal in the human body, Zn is naturally found in saliva and dental plaque at levels that, in theory, should not be limiting or toxic to bacteria. Yet, our recent characterization of the AdcABC Zn import system suggested that Zn might be a growth-limiting factor in dental biofilms, as inactivation of the *adcABC* metal transport system greatly reduced *S*. *mutans* colonization in a rat oral infection model [38]. Whether this apparent Zn restriction was caused by host-mediated Zn sequestration mechanisms, increased competition for environmental Zn with microbial commensals, or the combination of both remains to be determined. At the other end of the spectrum, Zn is required for host immune function and shows antimicrobial properties at elevated concentrations, such that it is harnessed by the host to intoxicate invading pathogens within phagocytic cells and, for centuries, has been used as a therapeutic agent to treat a variety of human conditions due to its antimicrobial and anti-inflammatory properties. In oral health, Zn is incorporated into oral healthcare products to treat gingivitis and to control halitosis [27,28] though its usefulness to caries treatment or prevention has proven to be controversial [27,33,53,54]. In this study, we used transcriptomic and molecular genetic approaches to uncover the mechanisms that allow *S*. *mutans* to overcome Zn toxicity. Through RNA-Seq analysis, we identified a previously uncharacterized P-type ATPase transporter, encoded by *smu2057c* (*zccE*), as involved in Zn tolerance. Importantly, we discovered that *zccE* is part of the core genome and unique to *S*. *mutans*. By comparing Zn tolerance levels of several streptococcal species that do not encode ZccE with those of the wild-type and Δ*zccE* strains of *S*. *mutans*, it became clear that ZccE was directly responsible for the inherently high Zn tolerance of *S*. *mutans*. Like other bacterial metal-translocating P-type ATPases that have the capacity to translocate multiple metals, loss of *zccE* also led to increased sensitivity to Cd, Co, and Cu. In addition, we identified *zccR* (*smu2058*), a member of the MerR family of transcriptional factors, as the major transcription factor controlling *zccE* expression in a Zn-dependent manner.

In addition to identification of *zccE*, the RNA-Seq analysis provided additional clues on how *S*. *mutans* might overcome Zn intoxication. For example, transcriptional changes in the expression of sugar transport and sugar metabolism genes suggest that *S*. *mutans* activates the tagatose-6-phosphate pathway to bypass the initial steps of the glycolytic pathway. These steps have been shown to be inhibited by millimolar concentrations of Zn in the closely related species *S*. *pyogenes* (Ong, Walker, & McEwan, 2015). Specifically, our transcriptome results revealed that elevated Zn concentrations repressed transcription of PTS genes involved in glucose and glucose disaccharide metabolism whereas genes of the lactose PTS and the tagatose pathway were strongly induced (**S1 Table and Fig 2**). A well-known mechanism utilized by bacteria to overcome Zn toxicity is based on the accumulation of Zn-buffering molecules including cysteine-containing molecules such as glutathione and free cysteine [8,55]. Our RNA-Seq analysis also revealed that *S*. *mutans* upregulates expression of genes involved in glutathione transport (*tcyABC*) and regeneration (*gst*), cystine transport (*tcyDEFG*) and metabolism (*cysK*) in response to high Zn stress. Future studies are necessary to test the hypothesis that *S*. *mutans* activates lactose metabolism to bypass a possible metabolic bottleneck created by Zn-mediated inhibition of the initial steps of glycolysis, and to whether glutathione and cysteine are indeed used as Zn-buffering systems to protect *S*. *mutans* against Zn intoxication.

Despite the multiple strategies bacteria utilize to mitigate Zn poisoning, it is the capacity to efficiently pump Zn out of their cytosol that seems to primarily define the levels of Zn tolerance. Until now, the only Zn efflux system known in streptococci was encoded by *czcD*, a CDF-type efflux system that has been characterized in major human pathogens including *S*. *pneumoniae*, *S*. *pyogenes*, and *S*. *agalactiae* [9,18,19,22]. However, the genome of *S*. *mutans* does not encode a *czcD* homologue; the only *S*. *mutans* gene coding for a CDF-type transporter is *smu1176* (*mntE*), which was recently shown to alleviate Mn toxicity [56]. Of interest, several bacteria, including the Gram-positive paradigm *B*. *subtilis*, have been shown to utilize both a CDF-type transporter and a P-type ATPase transporter to maintain Zn homeostasis during high Zn stress [12,14].

P-type ATPases are a large family of transmembrane proteins that use the energy generated by ATP hydrolysis to transport cations and other substrates across membranes. P_1B_-type ATPases are primarily involved in metal ion transport and are comprised of six to eight transmembrane helices that form the ionic channel across the cell membrane with two soluble cytoplasmic domains. In these metal translocating P-type ATPases, one of the eight helices bears a tripeptide ‘CPC’ metal-binding signature motif with a conserved center proline flanked by either cysteine/serine or histidine at either side [(C**/**S/T)P(C**/**H/P)] [57,58]. While the proline residue is essential, the amino acids surrounding the proline have been shown to define metal specificity of these transmembrane proteins. For example, the canonical *E*. *coli* ZntA has a fully conserved ‘CPC’ motif that confers high selectivity to Pb followed by Zn and Cd, and low selectivity to Co, Cu and Ni – substitution of one of the surrounding cysteines by histidine or serine resulted in loss of binding to Pb but not to Zn or Cd [59]. Based on previous studies, P_1B_-type ATPases with ‘CPC’ motifs are selective for silver (Ag), Cd, Cu, Pb, and Zn whereas proteins with SPC or CPH motifs are associated with Co and Cu export [59]. In addition to the ‘CPC’ motif, metal specificity is determined by the presence of GxxCxxC motif at the N-terminal end and the presence of a preceding aspartate residue [59]. Although ZccE possesses an ‘SPC’ motif, the N-terminal GxxCxxC motif is absent in ZccE such that the coordination chemistry and key residues of ZccE that determine metal specificity or promiscuity remain to be fully determined.

One of the best described mechanisms by which Zn exerts its toxicity in bacteria is by impairing Mn homeostasis [18,60]. In *S*. *pneumoniae*, Zn binds to the Mn transporter PsaABC when present at high concentrations, competitively affecting Mn uptake and rendering *S*. *pneumoniae* hypersensitive to oxidative stress and immune cell killing [61]. Of note, Zn outcompetes Mn for binding to PsaABC without being internalized [61]. Follow up studies with *S*. *pneumoniae* and *S*. *agalactiae* indicated that a drastic alteration in Zn:Mn ratios, rather than Zn concentration itself, was the main cause for Zn toxicity; Mn supplementation alone restored the equilibrium of Zn:Mn ratios and alleviated the growth defect phenotypes associated with high Zn stress [22,60,61]. Here, we found that the ~10-fold increase in intracellular Zn levels in Δ*zccE* was accompanied by a similar decrease in intracellular Mn pools (**Fig 7**), such that Zn:Mn ratios changed from ~1:1 before addition of Zn to ~100:1 after the challenge (**S9 Fig**). While Zn has been shown to compete with Mn for binding to the DtxR family transcriptional repressor SloR [62], our RNA-Seq analysis indicates that the decrease in total Mn pools was not caused by altered transcription of the SloR-regulated genes responsible for either Mn import (*sloABC* and *mntH*) or export (*mntE*) [50,56]. Mn plays multiple and important roles in oxidative stress tolerance, so this notable decrease in intracellular Mn pools provides one possible explanation, albeit likely not the only one, for the hypersensitivity of Δ*zccE* to H_2_O_2_ when simultaneously exposed to sub-inhibitory concentrations of Zn (**Fig 9**). To check whether the Zn:Mn ratio is also affected at the sub-inhibitory concentration of Zn that was used in the H_2_O_2_ sensitivity experiments, we also determined the intracellular Zn and Mn concentrations and Zn:Mn ratios in parent and Δ*zccE* cells grown in 150 μM Zn. While not as robust as the differences observed in media containing 1 mM Zn, Zn:Mn ratios were significantly altered when the Δ*zccE* strain was grown in media containing 150 μM Zn (**S9 Fig**). Of interest, the parent strain was capable of maintaining steady levels of both Zn and Mn (and therefore balanced Zn:Mn ratios) when challenged with either 150 or 1 mM Zn, suggesting that intracellular and not extracellular Zn levels are affecting Mn homeostasis. To further explore the association of Mn with Zn toxicity effects, we asked if Mn supplementation could rescue phenotypes of the *ΔzccE* strain when exposed to excess Zn. When *ΔzccE* was exposed to high Zn stress, the addition of Mn offered only minimal growth restoration (**S10 Fig**). However, the restorative effect of Mn supplementation was clear in the context of tolerance of Δ*zccE* to H_2_O_2_ (**S10 Fig**). Taken together, these results support previous investigations that demonstrate that Zn and Mn homeostasis are intertwined, and that this relationship is critical to bacterial pathophysiology. For this reason, mechanistic studies to understand how intracellular Zn perturbs Mn flux in *S*. *mutans* warrants further investigation.

To explore the potential of ZccE as an antimicrobial target, we performed an oral colonization study that included topical application of a ZnSO_4_ treatment to animals that had been infected with *S*. *mutans* UA159 or Δ*zccE*. As anticipated, the Δ*zccE* strain was susceptible to Zn treatment whereas the parent strain was not (**Fig 10**). These results suggest that targeted inhibition of ZccE expression or activity can be combined with Zn to develop an anti-caries therapy by specifically killing *S*. *mutans*.

**Fig 11:**
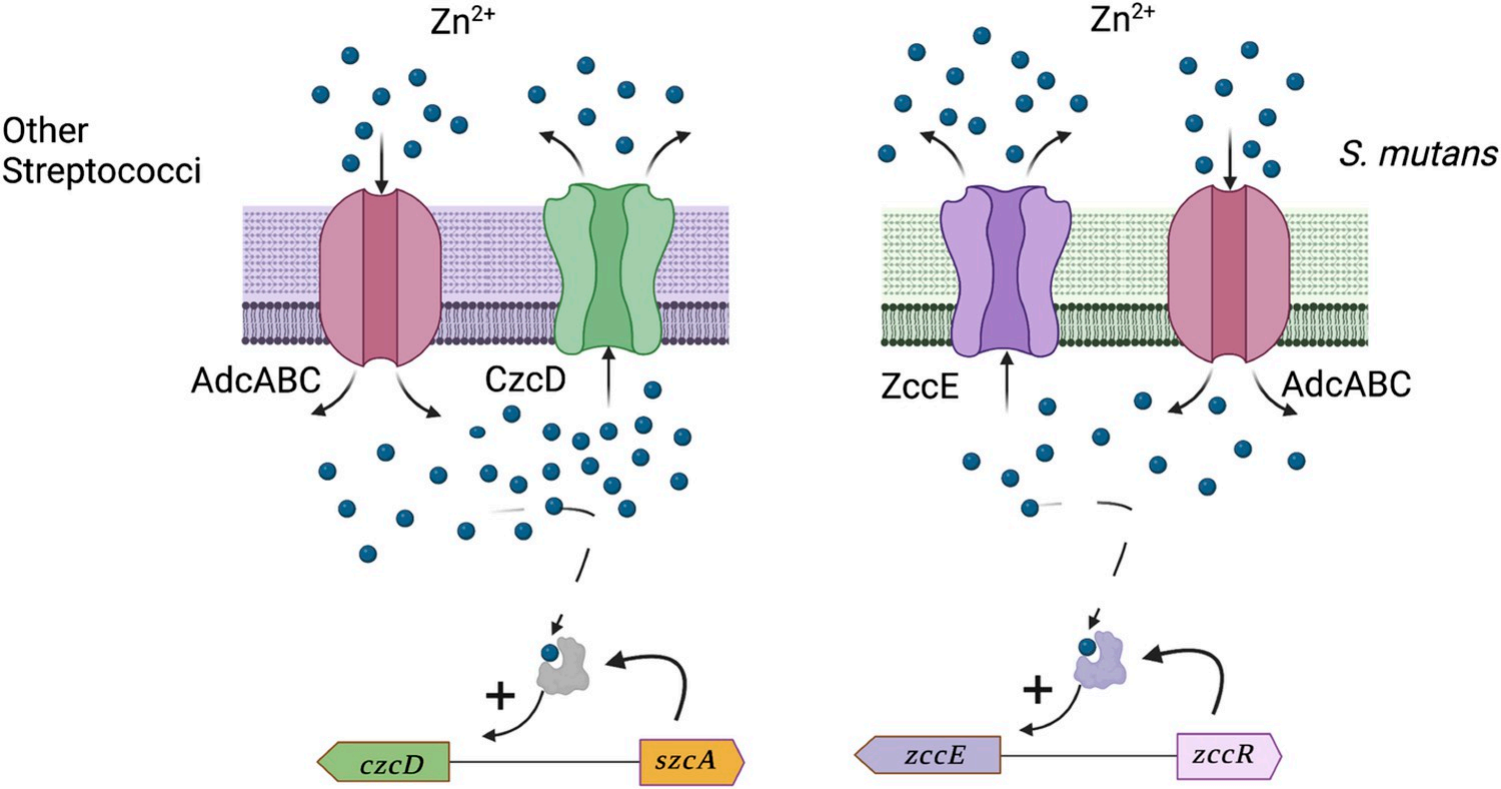
Model figure showing that ZccE (a P-type ATPase transporter) and CzcD (a CDF-type transporter) mediate high Zn tolerance in streptococci. Both *czcD* and *zccE* encode for Zn efflux systems and each system is positively regulated by cognate transcriptional regulators (SzcA for *czcD* and ZccR for *zccE*) that are activated under high Zn conditions. The CzcD/SzcA pair is found in most streptococci while ZccE/ZccR is unique to *S*. *mutans* and, based on Zn sensitivity assays and mutational analysis, is directly responsible for the exceptionally high Zn tolerance of *S*. *mutans* when compared to other streptococcal species. AdcABC is an ABC-type transporter conserved among streptococci and primarily responsible for Zn import. This figure was created with Biorender.

To summarize, this report sheds new light onto the molecular mechanisms utilized by *S*. *mutans* to overcome Zn toxicity. We discovered that *S*. *mutans* is inherently more tolerant to the toxic effects of Zn than other streptococci, including several non-cariogenic oral streptococci that are associated with oral health. We also identified the major effectors of high Zn tolerance in *S*. *mutans*, the metal translocating P_1B_-type ATPase ZccE and the Zn-responsive transcriptional factor ZccR. As described in **Fig 11**, while Zn import in streptococci is mediated mainly by a highly conserved ABC type transporter AdcABC, the Zn export in *S*. *mutans* differs significantly from other streptococcal species by utilizing a unique Zn exporter ZccE that provides *S*. *mutans* a clear survival advantage over other streptococci in high Zn environment. Because ZccE is unique to *S*. *mutans*, we propose that small molecules that specifically inhibit ZccE activity (or expression) can be combined with a Zn source to kill *S*. *mutans*. Thus, the combination of a ZccE-specific inhibitor with Zn can be exploited as a new antimicrobial therapy to prevent dental caries or to treat systemic *S*. *mutans* infections.

## Materials and methods

### Ethics statement

Animal procedure for rat oral colorizations was approved by the University of Florida Institutional Animal Care and Use Committee (protocol # 201810421). All animal care was consistent with the Guide for the Care and Use of Laboratory Animals from the National Research Council and the USDA Animal Care Resource Guide.

### Bacterial strains and growth conditions

The strains used in this study are listed in **Table 2**. All strains were routinely grown in brain heart infusion (BHI) at 37°C in a 5% CO_2_ atmosphere. When appropriate, antibiotics were added to cultures at the following concentrations: spectinomycin (1 mg ml^-1^), kanamycin (1 mg ml^-1^), erythromycin (10 μg ml^-1^). BHI and the chemically-defined medium FMC [63] were used to generate growth curves using an automated growth reader (Bioscreen C; Oy Growth Curves Ab, Ltd.). Briefly, overnight cultures were sub-cultured 1:20 in fresh media, grown to mid-exponential phase (OD_600_ of 0.4), and diluted 1:50 into the appropriate medium with or without metal supplementation in the wells of a microtiter plate. An overlay of sterile mineral oil was added to each well to minimize generation of reactive oxygen species. For RNA-Seq and qPCR analyses, bacterial cultures were grown in BHI to mid-log phase (OD_600_ of 0.4, untreated control) and then exposed to a final Zn concentration of 4 mM (using a concentrated ZnSO_4_ solution) for 15 or 90 minutes.

**Table 2:** Bacterial strains used in the study.

Strains	Relevant characteristics	Source
*S*. *mutans* UA159	Wild-type	Lab collection
*S*. *mutans* OMZ175	Wild-type	Lab collection
*S*. *mutans* LAR01	Wild-type	Lab collection
*S*. *mutans* 19C1	Wild-type	Lab collection
*S*. *mutans* 29SS	Wild-type	Lab collection
*S*. *sobrinus* SL1	Wild-type	Lab collection
*S*. *sobrinus* 6715	Wild-type	Lab collection
*S*. *sobrinus* B13	Wild-type	Lab collection
*S*. *gordonii* DL1	Wild-type	Lab collection
*S*. *gordonii* BCC29	Wild-type	Lab collection
*S*. *sanguinis* BCC46	Wild-type	Lab collection
*S*. *sanguinis* SK36	Wild-type	Lab collection
*S*. *intermedius* SK54	Wild-type	Lab collection
*S*. *oralis* SK92	Wild-type	Lab collection
*S*. *mitis* S-3	Wild-type	Lab collection
*S*. *agalactiae* A909	Wild-type	Lab collection
*S*. *pyogenes* NZ131	Wild-type	Lab collection
*S*. *mutans* Δ*zccE*	*zccE*::Spec^R^	This study
*S*. *mutans* OMZΔ*zccE*	*zccE*::Spec^R^	This study
*S*. *mutans ΔzccR*	*zccR*::Spec^R^	This study
*S*. *mutans ΔcopYAZ*	*copYAZ*::Erm^R^	This study
*S*. *mutans* Δ*zccE*Δ*copYAZ*	*zccE*::Spec^R^; *copYAZ*::Erm^R^	This study
*S*. *mutans ΔzccE*^*comp*^	*zccE*::Spec^R^, *zccE*+::Kan^R^	This study
*S*. *mutans* Δ*copYAZ*^comp^	*copYAZ*::Erm^R^, *copYAZ*+::Kan^R^	This study
*S*. *mutans ΔzccR*^comp^	*zccR*::*S*pec^R^, *zccR*+::Kan^R^	This study
*S*. *mutans* UA159-Km	Wild-type (Kanamycin resistance cassette in *gtfA* locus)	Gift from Zheng lab [64]
*E*. *coli* DH10B + pET30c	pET-30c (+)	Lab collection
*E*. *coli* HST08 Stellar Competent Cells (SCC)	Chemically competent cells	TaKaRa
*E*. *coli* BL21 λDE3		Lab collection
*E*. *coli* SCC + pET30c-*zccR*	pET30c-*zccR*+	This study
*E*. *coli* BL21 + pET30c-*zccR*	pET30c-*zccR*+	This study

### Construction of mutant and complemented strains

Strains lacking *zccE*, *zccR*, or the *copYAZ* operon were constructed using a standard PCR ligation mutagenesis approach [65]. Briefly, DNA fragments (~ 1 kb) flanking the target gene were amplified by PCR, digested with restriction enzymes, and ligated to a similarly-digested nonpolar spectinomycin resistance cassette for constructing Δ*zccE* and Δ*zccR* strains and to a nonpolar erythromycin resistance cassette to obtain the Δ*copYAZ* strain. The ligation mixtures were used to transform *S*. *mutans* UA159 in the presence of XIP (*com**X*-inducing peptide) following an established protocol [66]. Mutant strains were selected on BHI agar supplemented with the appropriate antibiotic and gene deletions confirmed by PCR amplification and Sanger sequencing. The double Δ*zccE*Δ*copYAZ* mutant was obtained by transforming the Δ*copYAZ* strain with the *zccE*::Spec^R^ region. To generate complemented strains, the full length *zccE*, *zccR*, or *copYAZ* coding sequences with their native promoters were amplified and cloned into the integration vector pMC340B [67] to yield plasmid pMC340B-*zccE*, pMC340B-*zccR*, and pMC340B-*copYAZ*, respectively. Plasmids were propagated in *E*. *coli* DH10B and used to transform the *S*. *mutans* strains Δ*zccE*, Δ*zccR*, or Δ*copYAZ* for integration at the mannitol utilization (*mtl*) locus. All primers used in this study are listed in **S2 Table**.

### Phylogenetic analysis

P-type ATPases, either experimentally characterized or predicted as metal exporters, were chosen to include at least two representative proteins from each characterized sub-family as well as proteins identified by NCBI’s BLASTP [68] to be relatively closely related (either in a search among streptococci or in search excluding all streptococci) [69]. Sequence labels were standardized and sequences were aligned using EMBL-EBI’s Clustal Omega [70] to generate a distance tree which was then modified for readability using the Interactive Tree of Life vs 6.5.8 [71].

### RNA analysis

Total RNA was isolated from homogenized *S*. *mutans* cell lysates by acid-phenol chloroform extractions as previously described [50]. Total RNA isolated was treated with Ambion DNase I (ThermoFisher) for 30 min at 37°C and further purified using an RNeasy kit (Qiagen), which included a second on-column DNase digestion. Sample quality and quantity were assessed on an Agilent 2100 Bioanalyzer at the University of Florida Interdisciplinary Center for Biotechnology Research (UF-ICBR). For RNA-Seq analysis, samples were prepared as previously described [50]. Briefly, 5 μg of the total RNA isolated was subjected to two rounds of mRNA enrichment using a MICROBExpress bacterial mRNA purification kit (Thermo Fisher), and 100 ng of the enriched mRNA used to generate cDNA libraries with unique barcodes (Next UltraII Directional RNA Library Prep kit for Illumina, New England Biolabs). The cDNA libraries were pooled and subjected to RNA-Seq analysis at the UF-ICBR using the Illumina NextSeq 500 platform. Read mapping was performed on a Galaxy server using Map with Bowtie for Illumina and the *S*. *mutans* UA159 genome (GenBank accession no. NC_004350.2) as a reference. The reads per open reading frame were tabulated with htseq-count. Final comparisons between control and Zn-treated conditions were performed with Degust (http://degust.erc.monash.edu/), with a false-discovery rate (FDR) of 0.05 and 2-fold change cutoff. Quantifications of *zccE* and *gyrA* mRNA were determined by qRT-PCR following an established protocol [72] using the primers listed in **S2 Table**. The fold change of *zccE* expression was performed using ΔΔ*C*_*T*_ (where *C*_*T*_ is the threshold cycle) method, with *gyrA* being used as standardization control. One-way ANOVA was performed to verify significance of the qRT-PCR results.

### Metal sensitivity assays

To test sensitivity to Zn in disc diffusion assays, 25 μl of exponentially grown cultures (OD_600_ ~ 0.5) were spread onto agar plates using a sterile swab and topped with sterile Whatman filter paper discs (6-mm diameter) saturated with 20 μl of a 1 mM ZnSO_4_ solution. The diameter of the zone of growth inhibition was measured after 24 h of incubation at 37°C in 5% CO_2_. The minimum inhibitory concentration (MIC) of ZnSO_4_ was determined by a broth microdilution method using two-fold serial dilutions of ZnSO_4_. Plates were incubated at 37°C in 5% CO_2_ for 16 h and the concentration of ZnSO_4_ at which the absorbance values were 10% of the control condition was determined to be the MIC. For plate titration assays, exponentially grown BHI cultures (OD_600_ ~ 0.5) were serially diluted and 8 μl of each 10-fold dilution spotted on BHI plates supplemented with selected trace metals. Plates were photographed after 24 h incubation at 37°C in 5% CO_2_ or under anaerobiosis (GasPak jar, BD Biosciences).

### Antagonism assay

The ability of *S*. *gordonii* to inhibit the growth of *S*. *mutans* via H_2_O_2_ production was assessed as described previously [50]. Briefly, 8 μl of an overnight culture of *S*. *gordonii* DL1 was spotted on BHI plates with or without Zn (50 μM ZnSO_4_) supplementation and incubated at 37°C in 5% CO_2_. After 24 h incubation, 8 μl of an overnight culture of *S*. *mutans* UA159 or Δ*zccE* was spotted proximal to the *S*. *gordonii* spot. To confirm that growth inhibition was due to H_2_O_2_ production, a control condition was included in which 8 μl of 1 mg ml^−1^ catalase solution was spotted on top of the *S*. *gordonii* spot prior to spotting *S*. *mutans*. To obtain quantitative data of the *S*. *mutans*-*S*. *gordonii* competition, *S*. *mutans* strains were either co-cultured with *S*. *gordonii* DL1 or grown alone for 24 hours in BHI media containing 0, 0.05, or 0.1 mM Zn before plating on media selective for *S*. *mutans*. BHI agar supplemented with 1 mg/ml of kanamycin was used for selection of UA159-Km, a derivative of the parent strain UA159 for which a kanamycin resistance gene has been incorporated at the *gtfA* locus [64]. BHI agar supplemented 1 mg/ml of spectinomycin was used for selection of the *ΔzccE* strain.

### ICP-MS analysis

The intracellular Zn and Mn content in parent and mutant strains was determined via inductively coupled plasma mass spectrometry (ICP-MS) performed at the University of Florida Institute of Food and Agricultural Sciences (UF-IFAS) Analytical Services Laboratories. Briefly, cultures (250 ml) were grown in BHI to mid-exponential phase (OD_600_ 0.4), harvested by centrifugation at 4°C for 15 min at 4,000 rpm, washed in PBS supplemented with 0.2 mM EDTA (to chelate extracellular divalent cations) followed by a second wash in PBS only. The cell pellets were resuspended in 35% (v/v) HNO_3_, heated at 95°C for 1 h before diluted to 3.5% (v/v) HNO_3_ using metal free water and the intracellular Zn and Mn content determined using a 7900 ICP Mass Spectrometer (Agilent). Metal concentrations were normalized to total protein content determined by the bicinchoninic acid (BCA) assay (Pierce).

### ZccR purification

To overexpress and purify a recombinant His-tagged ZccR, the 795-bp *zccR* coding region was amplified by PCR using the primers listed in **S2 Table**, and the amplicon cloned onto the expression vector pET30c (Novagen) using the In-fusion HD Cloning Plus kit (TaKaRa) to generate plasmid pET30c-*zccR*. The resultant plasmid was confirmed by Sanger sequencing and transformed into *E*. *coli* BL21 λDE3. The resulting *E*. *coli* strain harboring pET30c-*zccR* was grown in a shaking 37°C incubator in Luria-Bertani broth to mid-exponential phase (OD_600_ 0.4). Recombinant His-tagged ZccR was overexpressed by addition of 0.2 mM isopropyl-β-D-1-thiogalactopyranoside (IPTG) (Teknova) and the culture was incubated for an additional 3 h. Cell pellets were collected by centrifugation, resuspended in lysis buffer (50 mM NaH_2_PO_4_, 300 mM NaCl, 10 mM imidazole, pH 8) containing lysozyme (1 mg ml^-1^) and sonicated to clarity before centrifugation of cellular debris. The cleared lysate supernatant was mixed with pre-cleared Ni-NTA beads at 4°C for 60 minutes before loaded onto a chromatography column. The packed column was washed with wash buffer (50 mM NaH_2_PO_4_, 300 mM NaCl, 20 mM imidazole), and His-ZccR was eluted (50 mM NaH_2_PO_4_, 300 mM NaCl, 250 mM imidazole) according to the QIAexpressionist protocol (Qiagen) for purification of native proteins with histidine tags. The elution fractions were analyzed by 12% SDS-PAGE, and the fractions containing highly pure ZccR pooled and dialyzed against PBS. Aliquots of purified protein were stored at -20°C in 15% glycerol until further use.

### Electrophoretic mobility shift assays

EMSAs were performed with slight modifications from established protocols [50,73]. Briefly, the intergenic region (IGR) between *zccE* and *zccR* was amplified using the primers listed in **S2 Table**, and the resulting 160-bp amplicon was biotin-labeled using the Biotin 3’ End DNA Labeling kit (ThermoFisher). EMSA reactions were prepared in 20 μl reaction mixtures in binding buffer (10 mM Tris, 50 mM KCl, 1 μg poly dI-dC, 1 mM DTT; 5% glycerol, pH 7.5) containing 20 fmol of labeled probe and 0 to 1.4 μM of purified ZccR. Samples were loaded onto 6% non-denaturing polyacrylamide gels and resolved at 100V for 1h in cold buffer (4°C). Gels were transferred to BrightStar-Plus positively charged nylon membranes (Thermo-Fisher) and bands visualized using the Chemiluminescent Nucleic Acid Detection Module (Thermo-Fisher) following manufacturer’s protocol. To determine binding specificity, 100X molar excess of either non-labeled specific competitor DNA (*zccE*-*zccR* IGR) or non-specific competitor DNA (206-bp *S*. *mutans mntH* promoter region [50]) was added to the reaction.

### Oral colonization rat model

A modification of a rat caries model that we have previously followed [38] was used to determine the ability of the Δ*zccE* mutant strain to colonize rats fed a cariogenic diet while also testing the effects of topical Zn treatment on *S*. *mutans* colonization efficiency. Briefly, specific pathogen-free Sprague-Dawley rat pups were purchased with their dams from Envigo Laboratories and screened upon arrival to ensure an absence of mutans streptococci by plating oral swabbings on mitis salivarius (MS) agar. Prior to infection, pups and dams received 0.8 mg ml^-1^ sulfamethoxazole and 0.16 mg ml^-1^ trimethoprim in the drinking water for 3 days to suppress endogenous flora and facilitate colonization by *S*. *mutans*. After 4 days of washout during which antibiotic-free water was provided, dams and pups aged 18 days were orally infected for four consecutive days with actively growing *S*. *mutans* UA159 or Δ*zccE* cultures by means of cotton swab. At the time of infection, the regular chow diet was replaced by a 12% sucrose cariogenic powdered diet (ENVIGO diet TD.190707). During the infection period, the animals were provided with 5% (wt/vol) sterile sucrose-water *ad libitum*, then fresh water for the remainder of the study. On the final day of infection, pups were weaned and randomly placed into experimental groups. Topical treatment with ZnSO_4_ or saline solutions commenced after the fourth and final day of bacterial infection and lasted 10 consecutive days. Test (60 mM or 150 mM ZnSO_4_) or control (saline) treatments were topically administered to the rat teeth with a camel’s hair brush twice a day with a 6 h interval between treatments. At the end of the treatment period, animals were euthanized by CO_2_ asphyxiation, and the lower jaws removed for bacterial burden determination. Jaw sonicates were subjected to 10-fold serial dilutions and plated on MS agar (to count *S*. *mutans*) and 5% sheep blood agar (to count total flora). The number of *S*. *mutans* recovered from the animals was expressed as CFU ml-1 of jaw sonicate, and *S*. *mutans* colonies counted on MS agar divided by the total CFU on blood agar to determine the percentage of *S*. *mutans* colonies recovered over the total flora. This study was reviewed and approved by the University of Florida Institutional Animal Care and Use Committee (protocol # 201810421).

## Supporting information

S1 Fig Growth of selected streptococcal species in BHI or BHI containing 0.5 mM ZnSO_4_. Data represent means and standard deviations of results from at least 3 independent experiments.
(TIF)

S2 Fig Growth curves of *S*. *mutans* UA159 in the chemically-defined FMC medium spiked with 0, 2, 4 or 6 mM ZnSO_4_ upon reaching mid-logarithmic phase (OD_600_ ~ 0.3). Data represent means and standard deviations of results from at least 3 independent experiments.
(TIF)

S3 Fig Amino acid sequence alignment of *S*. *mutans* P-type ATPases Smu2057c (ZccE) and CopA and P-type ATPases that confer Zn tolerance to *B*. *subtilis* (CadA) and *E*. *coli* (ZntA). Blue shade depicts the N-terminal metal binding motif that is absent in ZccE, green shade depicts the metal binding ‘CPC’ motif, and the orange shade indicates the conserved phosphorylation site for ATPase activity.
(TIF)

S4 Fig Protein Structures of the P-type ATPases (A) ZccE, determined using AlphaFold2 and (B) ZntA, from PDB structure 4UMV, show that ZccE has a distinct structure among this group of ATPases. These structures were viewed through NCBI’s iCn3D. To distinguish secondary structure, sheets are depicted in yellow and helices in red. The metal specificity sites (SPC for ZccE and CPC for ZntA) depicts sidechains as sticks, highlighted in yellow and colored bright green. ZntA also includes the ligands associated with its PDB structure.
(TIF)

S5 Fig Growth of *S*. *mutans* OMZ175 and Δ*zccE* derivative in BHI with or without 1 mM ZnSO_4_ supplementation. Data represent means and standard deviations of results from at least 3 independent experiments.
(TIF)

S6 Fig Plate titration (spot test) of *S*. *mutans* UA159, Δ*zccE*, Δ*copYAZ* and Δcop*YAZ*Δ*zccE* strains on BHI plates supplemented with increasing concentration of CuSO_4_ and incubated for 24 h at 37°C under anaerobic conditions (Gaspack). Images are representative of at least 3 independent experiments.
(TIF)

S7 Fig Growth curves of UA159, Δ*zccE*, Δ*zccR* and respective complemented strains in BHI with or without Zn supplementation (150 μM ZnSO_4_). Data represent means and standard deviations of results from at least 3 independent experiments.
(TIF)

S8 Fig CFU measurement of *S*. *mutans* parent strain UA159, Δ*zccE* and the complemented strain grown in either the presence or absence of *S*. *gordonii* DL1 for 24 hours in BHI containing 0 mM, 0.05 mM and 0.1 mM of added Zn. Data represent average and standard deviation of values from three independent biological replicates. Two-way ANOVA was used to determine significance. A *p* value of <0.05 was considered significant (*).
(TIF)

S9 Fig ICP-MS quantifications of intracellular Zn (A) Mn (B) or Zn:Mn ratio (C) of *S*. *mutans* parent strain UA159 or isogenic mutant Δ*zccE* grown in BHI supplemented with 0mM, 0.15 mM, or 1 mM of Zn. Data represent the averages and standard deviations of values from at least three independent biological replicates. Two-way ANOVA was used to determine significance between metal content of either the same strain before and after Zn exposure or among different strains after Zn exposure. A *p* value of <0.05 was considered significant (*).
(TIF)

S10 Fig Growth of *S*. *mutans* UA159 and *ΔzccE* strains with Zn and Mn. (A) Growth in BHI medium with supplementation of Zn and Mn in different ratios. (B) Growth inhibition zones for *S*. *mutans* UA159 and Δ*zccE* strains grown on BHI agar containing 250 μM Mn with or without Zn supplementation and exposed to filter paper discs saturated with 0.25% H_2_O_2_. Data represent means and standard deviations of results from at least 3 independent experiments.
(TIF)

S1 Table RNA-Seq analysis of *S*. *mutans* UA159 genes differentially expressed when grown in FMC treated with 4 mM ZnSO_4_ for either 15 or 90 minutes and compared to untreated control. The genes whose expression changes over 2-fold applying False Discovery Rate (FDR) of 0.05 were mentioned and functional categories were listed.
(XLSX)

S2 Table The table lists the primers used in the study. The restriction enzyme sites that were incorporated into the primer sequences are shown in bold.
(DOCX)
