# Supplementary figures and images for: ZccE is a Novel P-type ATPase That Protects Streptococcus mutans Against Zinc Intoxication
Source: PLoS Pathog. 2022 Aug 8;18(8):e1010477. doi: 10.1371/journal.ppat.1010477 (PMC9387928; doi:10.1371/journal.ppat.1010477)

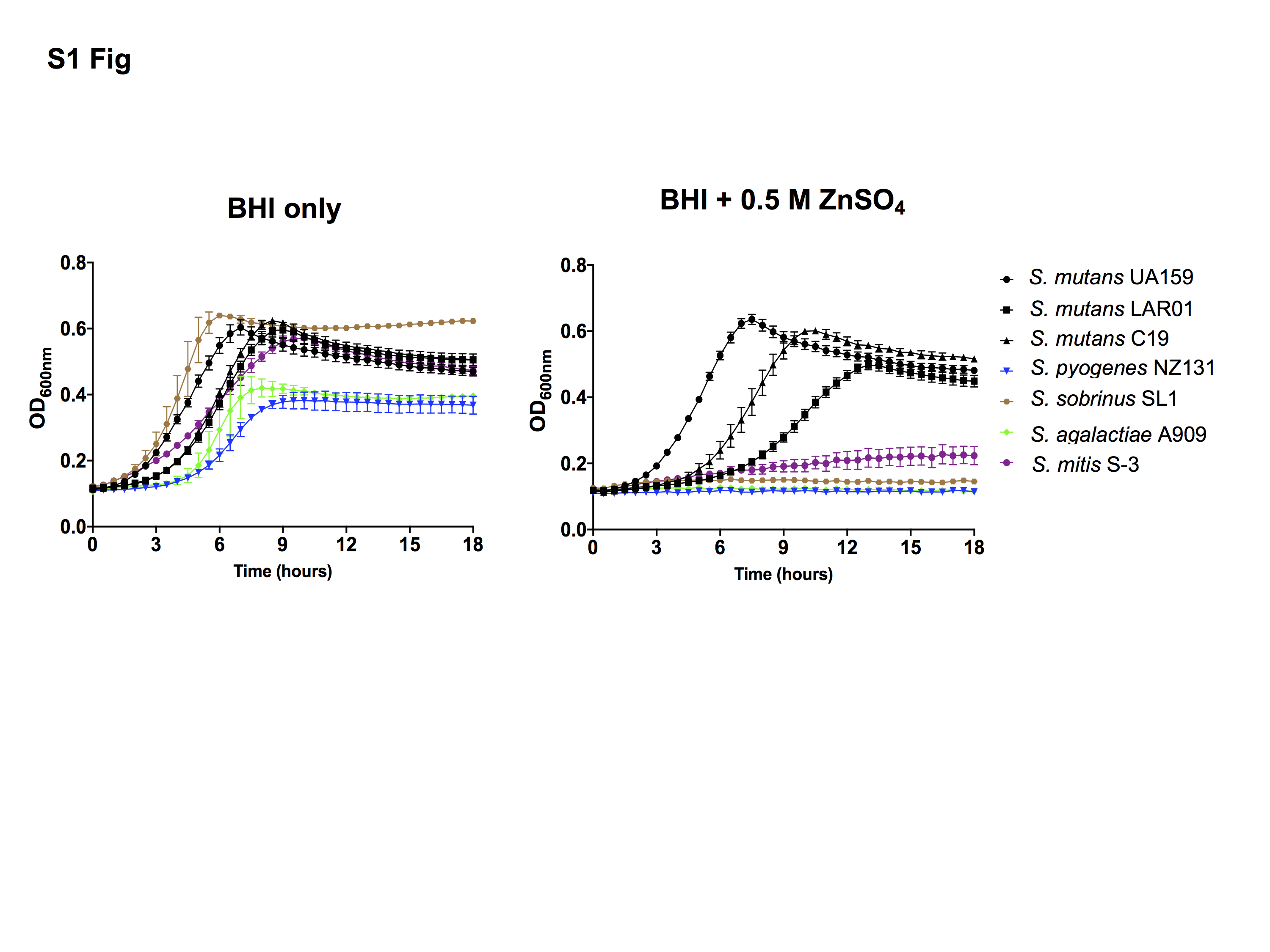

Supplement: S1 Fig — Data represent means and standard deviations of results from at least 3 independent experiments. (TIF) [file ppat.1010477.s001.tif]

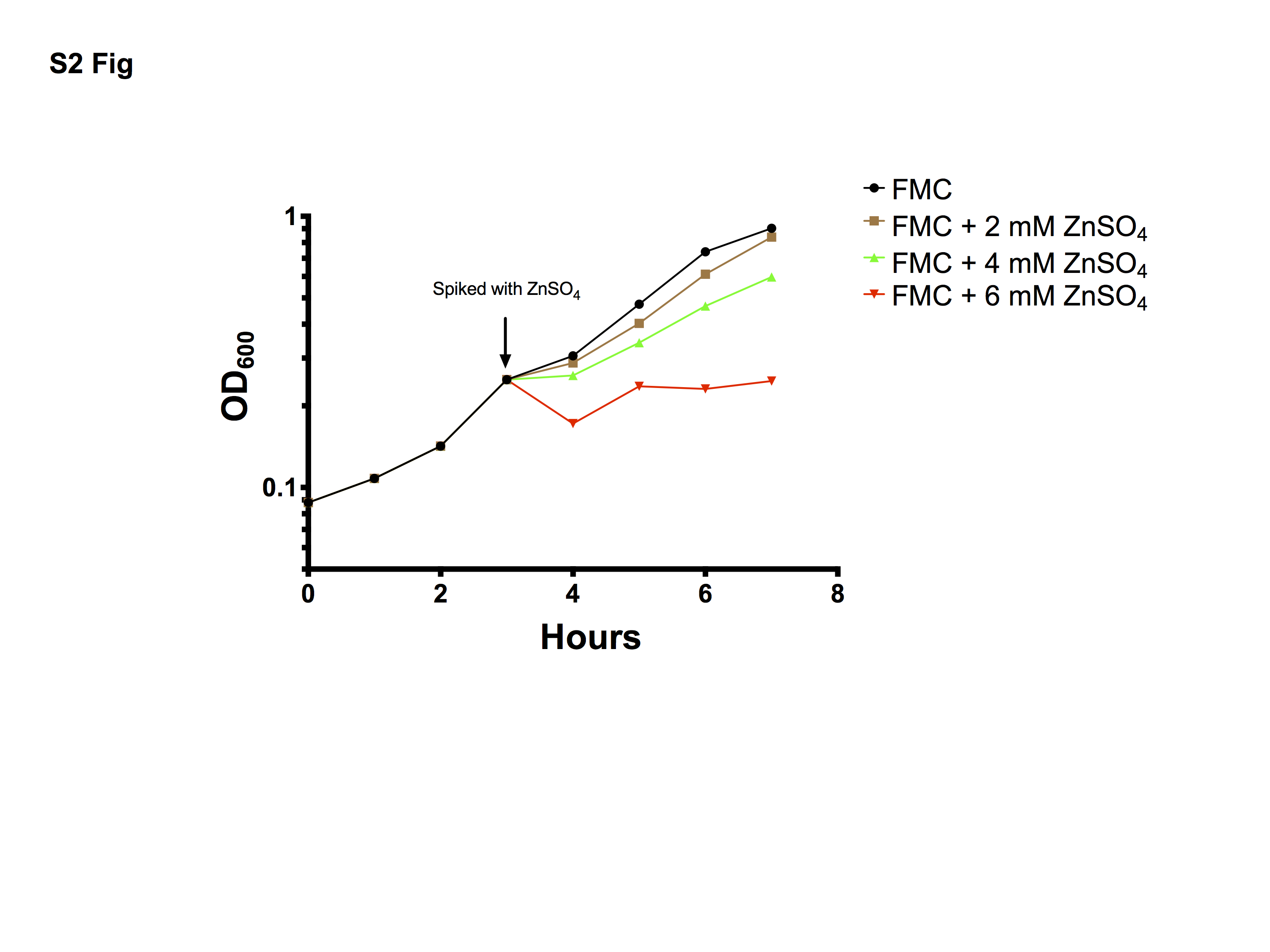

Supplement: S2 Fig — Data represent means and standard deviations of results from at least 3 independent experiments. (TIF) [file ppat.1010477.s002.tif]

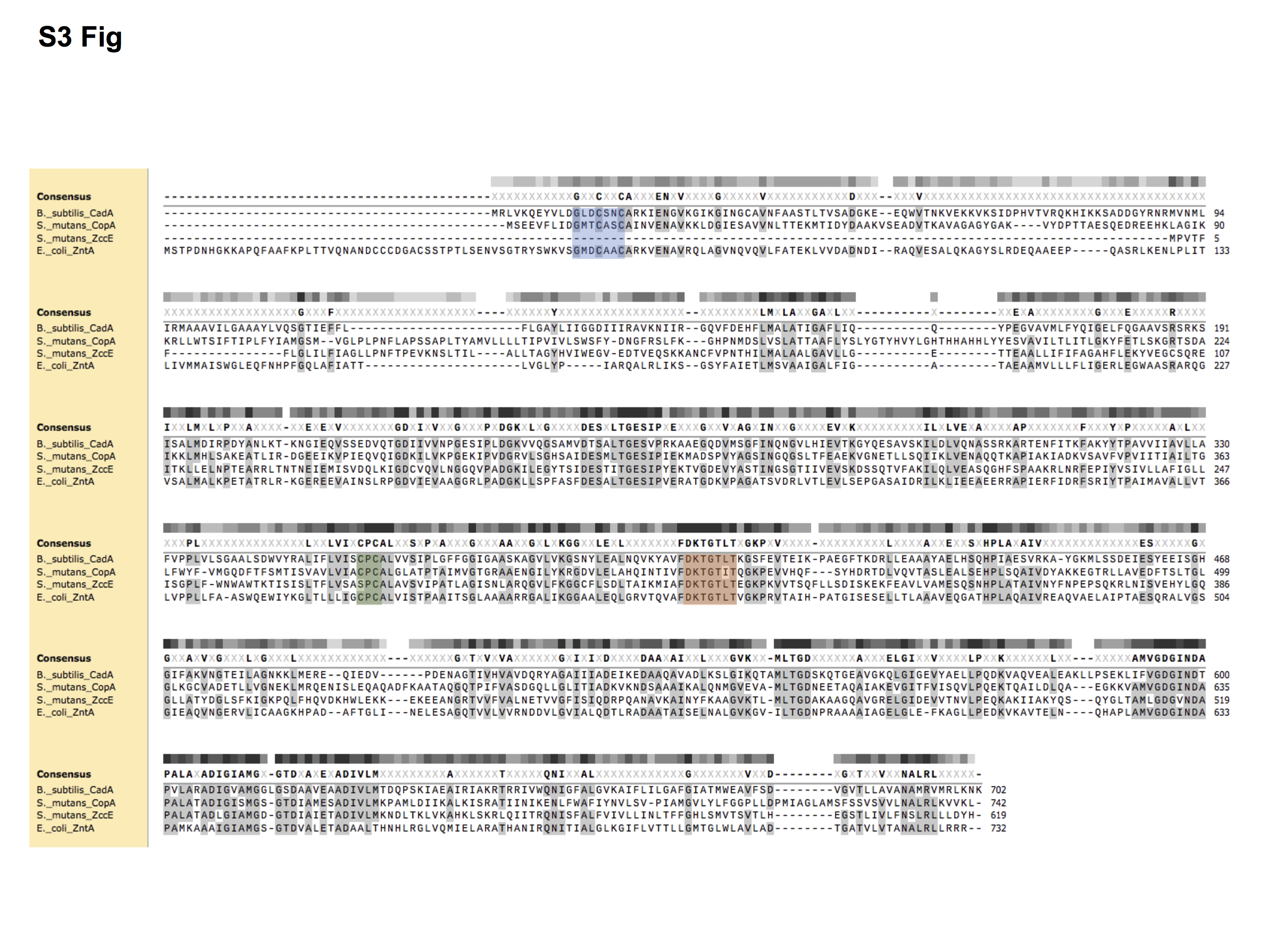

Supplement: S3 Fig — Blue shade depicts the N-terminal metal binding motif that is absent in ZccE, green shade depicts the metal binding ‘CPC’ motif, and the orange shade indicates the conserved phosphorylation site for ATPase activity. (TIF) [file ppat.1010477.s003.tif]

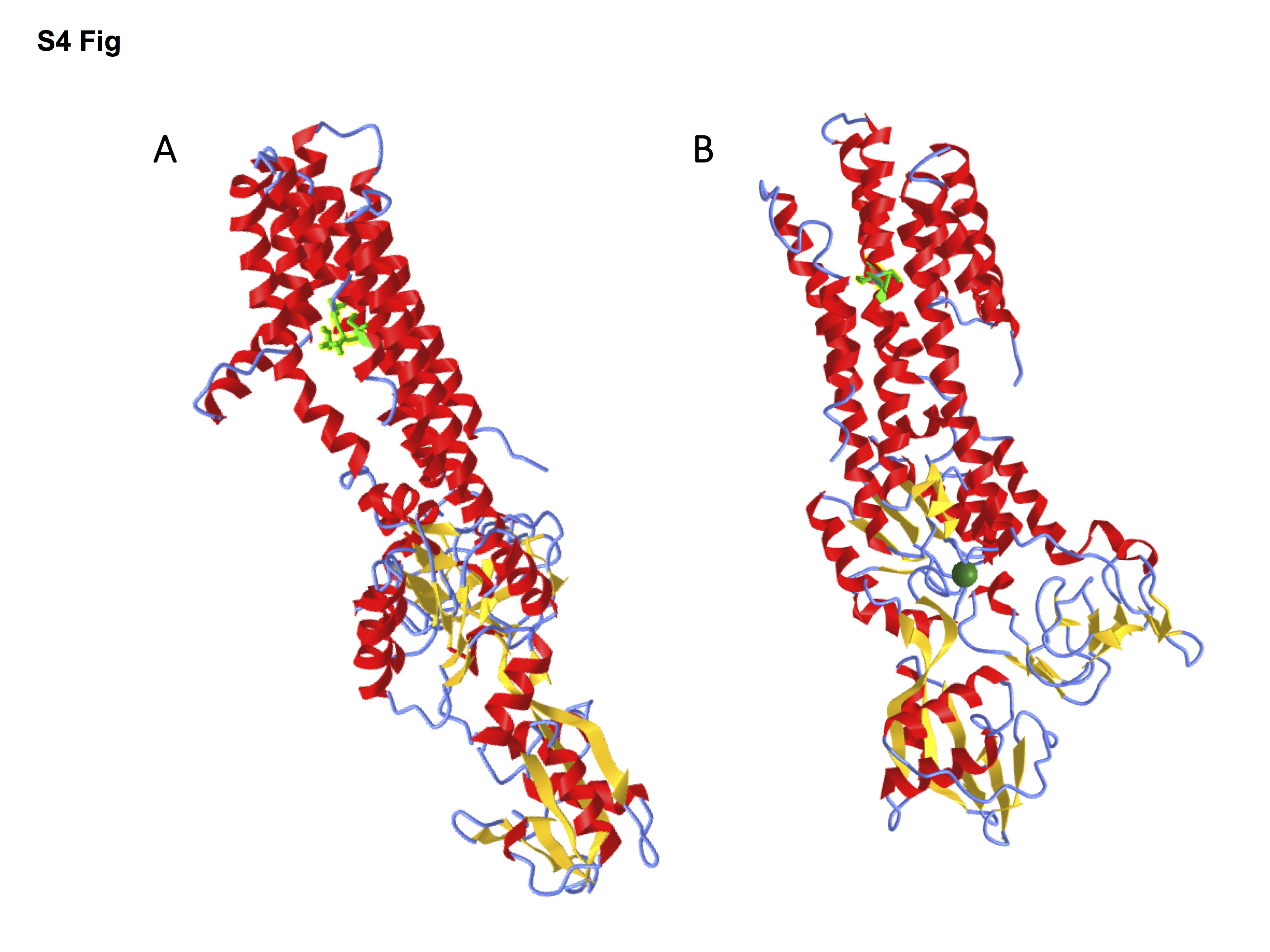

Supplement: S4 Fig — Protein Structures of the P-type ATPases (A) ZccE, determined using AlphaFold2 and (B) ZntA, from PDB structure 4UMV, show that ZccE has a distinct structure among this group of ATPases. These structures were viewed through NCBI’s iCn3D. To distinguish secondary structure, sheets are depicted in yellow and helices in red. The metal specificity sites (SPC for ZccE and CPC for ZntA) depicts sidechains as sticks, highlighted in yellow and colored bright green. ZntA also includes the ligands associated with its PDB structure. (TIF) [file ppat.1010477.s004.tif]

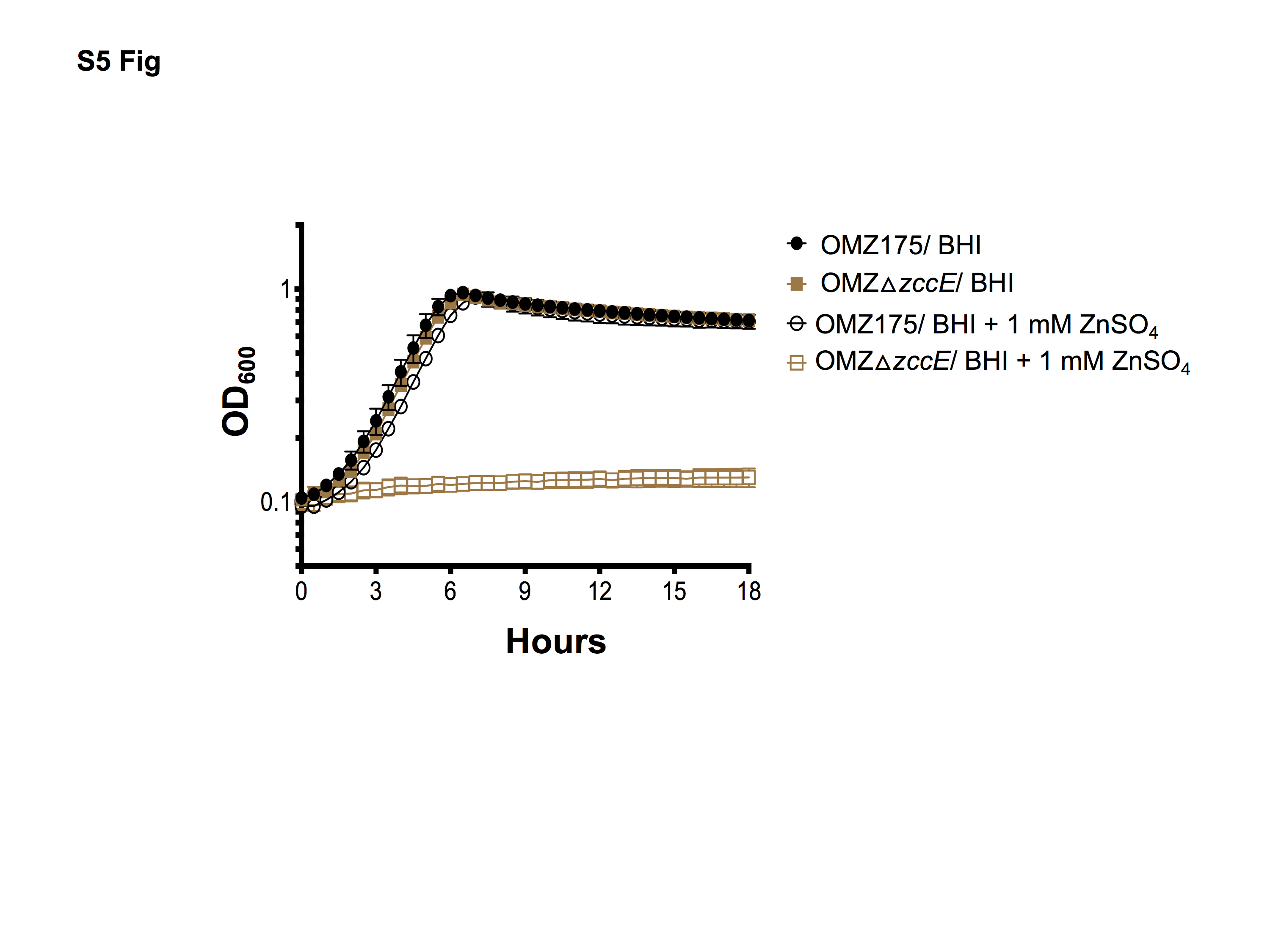

Supplement: S5 Fig — Data represent means and standard deviations of results from at least 3 independent experiments. (TIF) [file ppat.1010477.s005.tif]

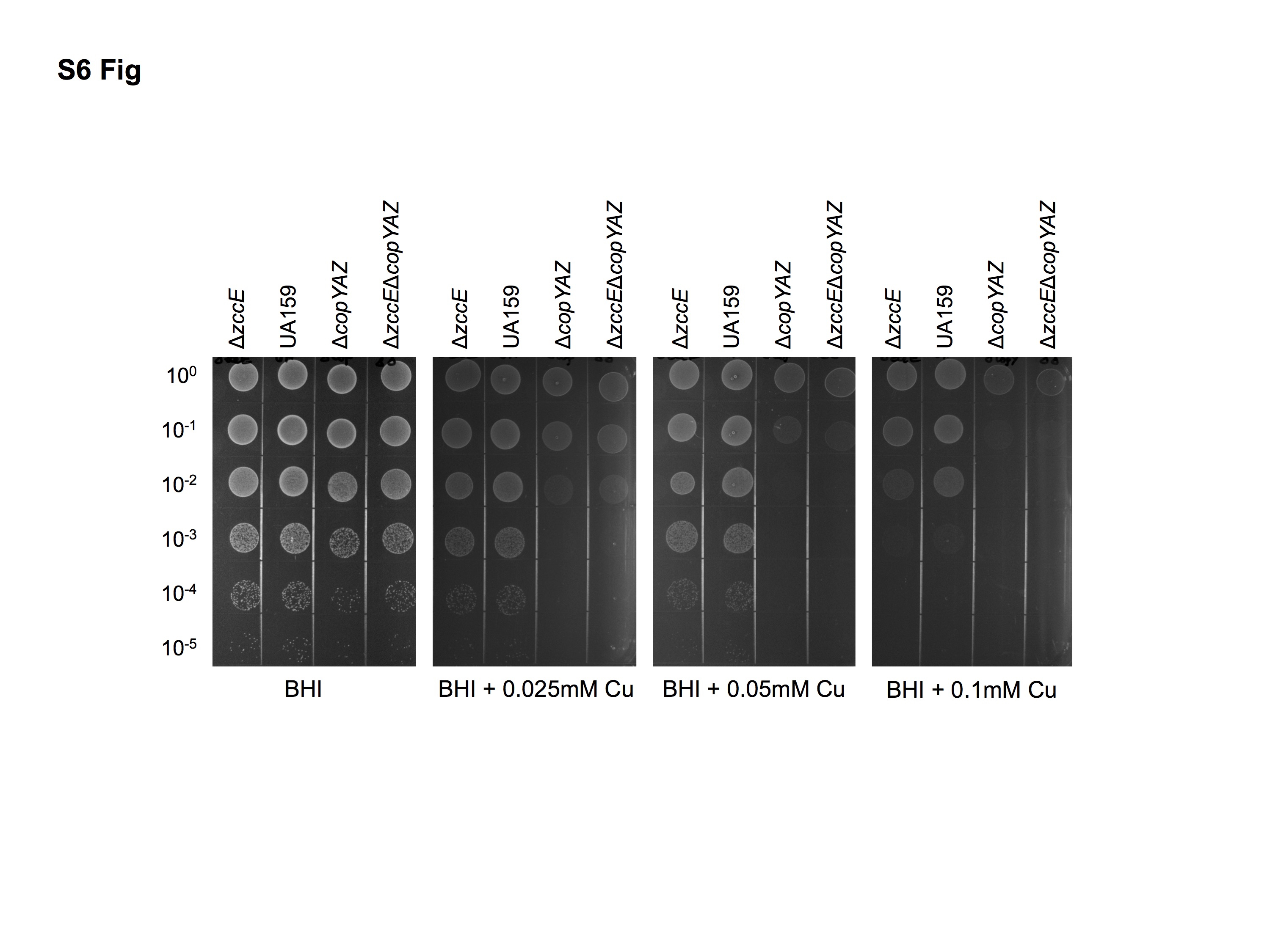

Supplement: S6 Fig — Images are representative of at least 3 independent experiments. (TIF) [file ppat.1010477.s006.tif]

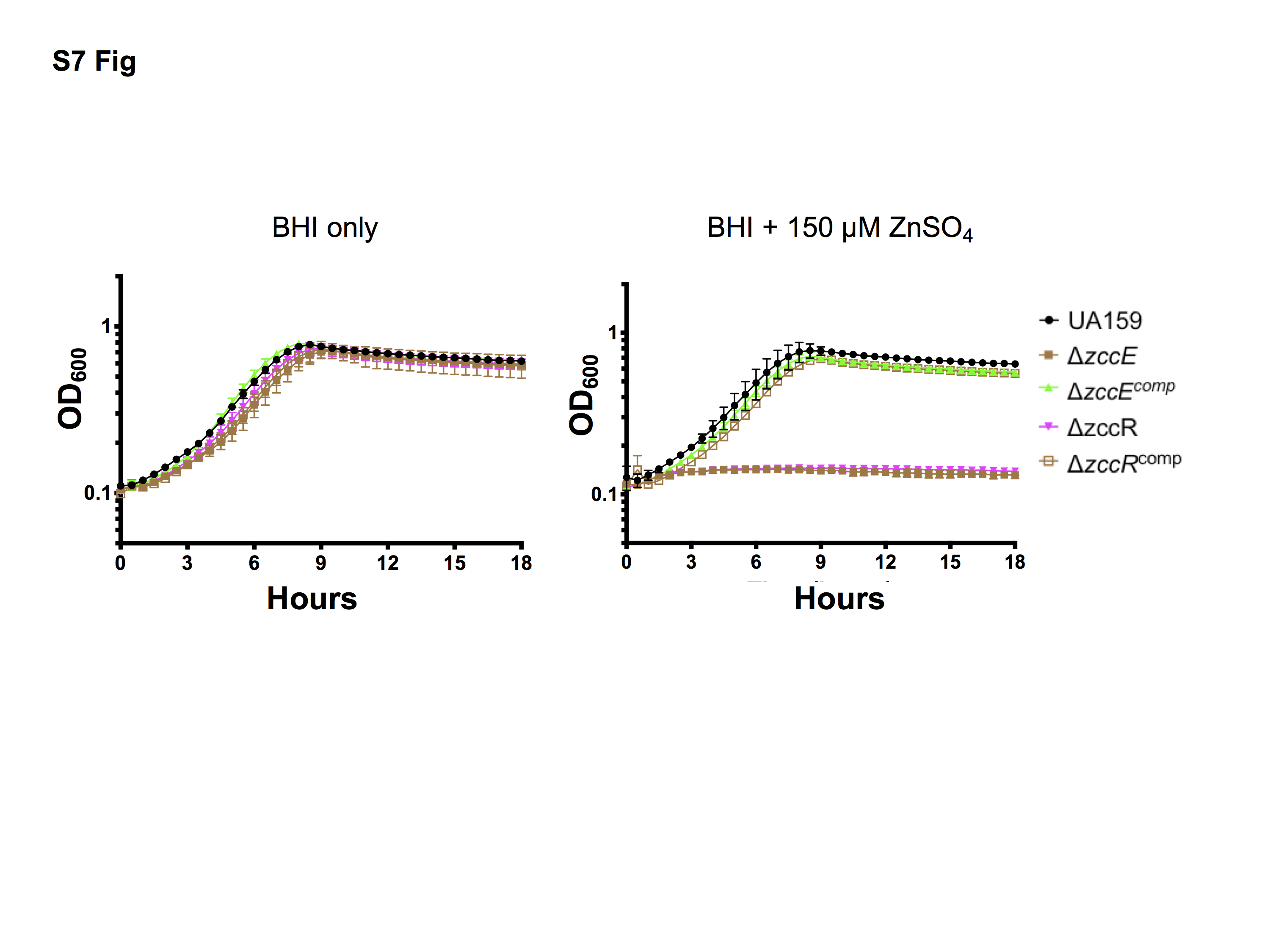

Supplement: S7 Fig — Data represent means and standard deviations of results from at least 3 independent experiments. (TIF) [file ppat.1010477.s007.tif]

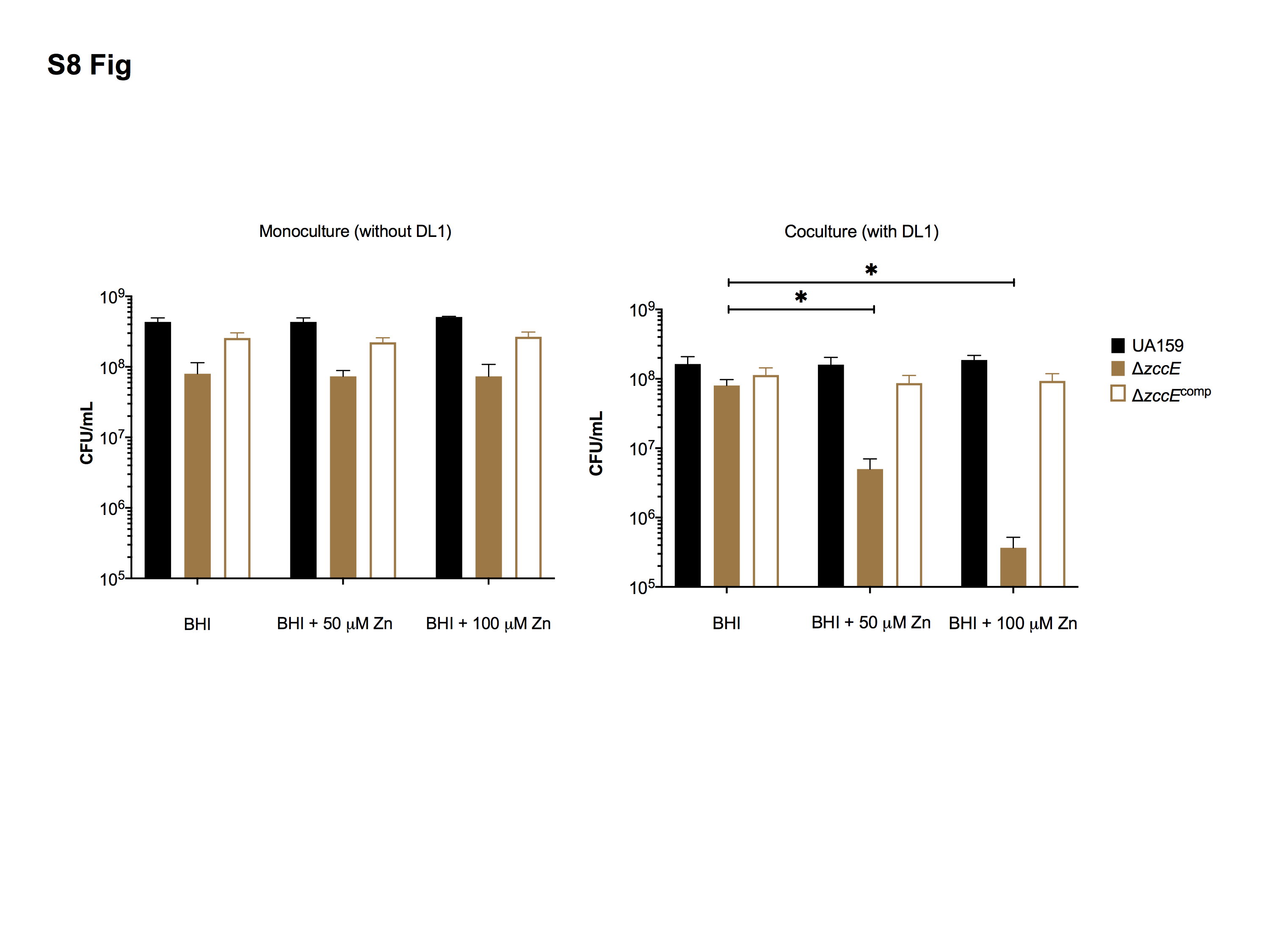

Supplement: S8 Fig — Data represent average and standard deviation of values from three independent biological replicates. Two-way ANOVA was used to determine significance. A p value of <0.05 was considered significant (*). (TIF) [file ppat.1010477.s008.tif]

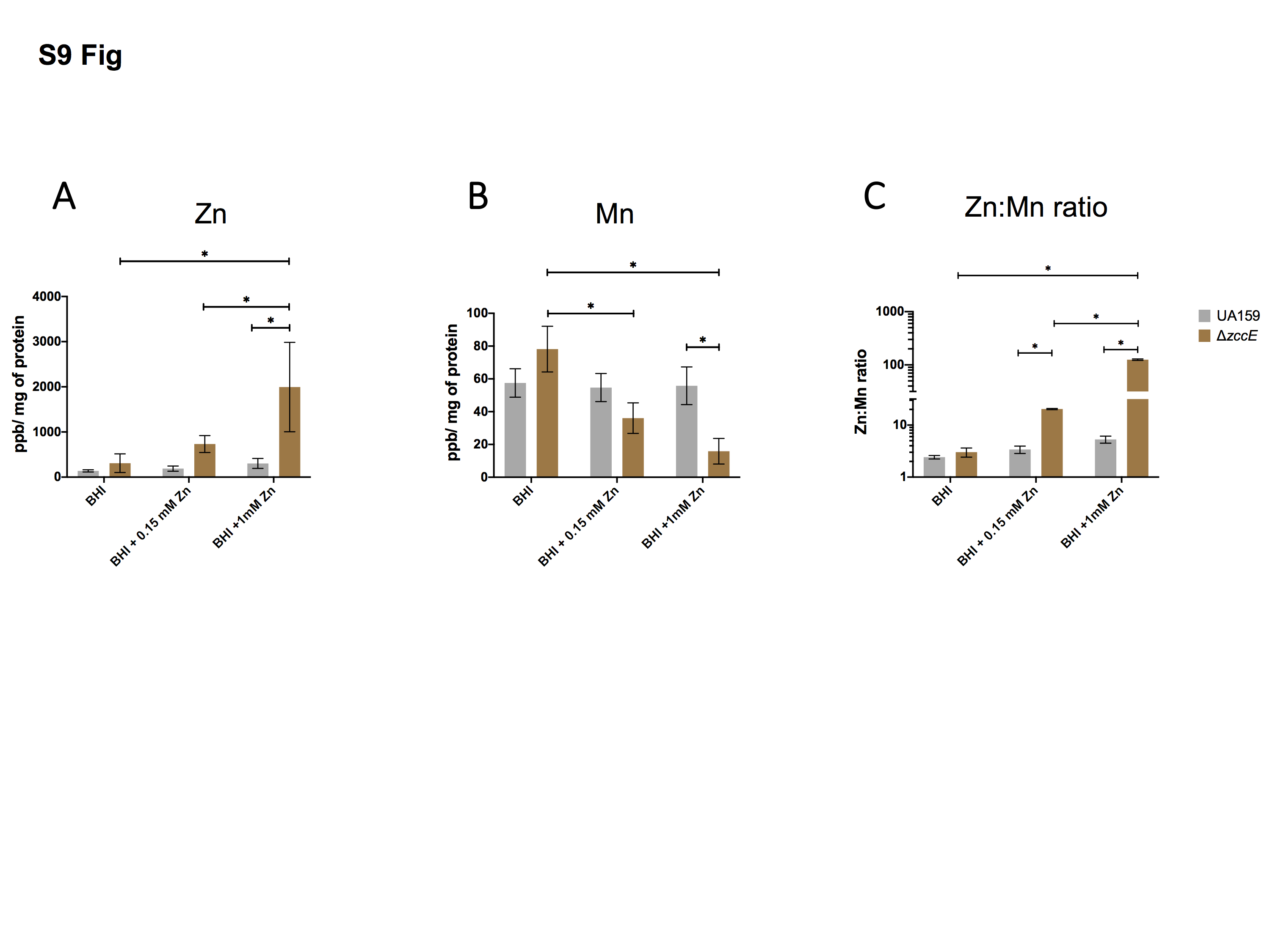

Supplement: S9 Fig — ICP-MS quantifications of intracellular Zn (A) Mn (B) or Zn:Mn ratio (C) of S. mutans parent strain UA159 or isogenic mutant ΔzccE grown in BHI supplemented with 0mM, 0.15 mM, or 1 mM of Zn. Data represent the averages and standard deviations of values from at least three independent biological replicates. Two-way ANOVA was used to determine significance between metal content of either the same strain before and after Zn exposure or among different strains after Zn exposure. A p value of <0.05 was considered significant (*). (TIF) [file ppat.1010477.s009.tif]

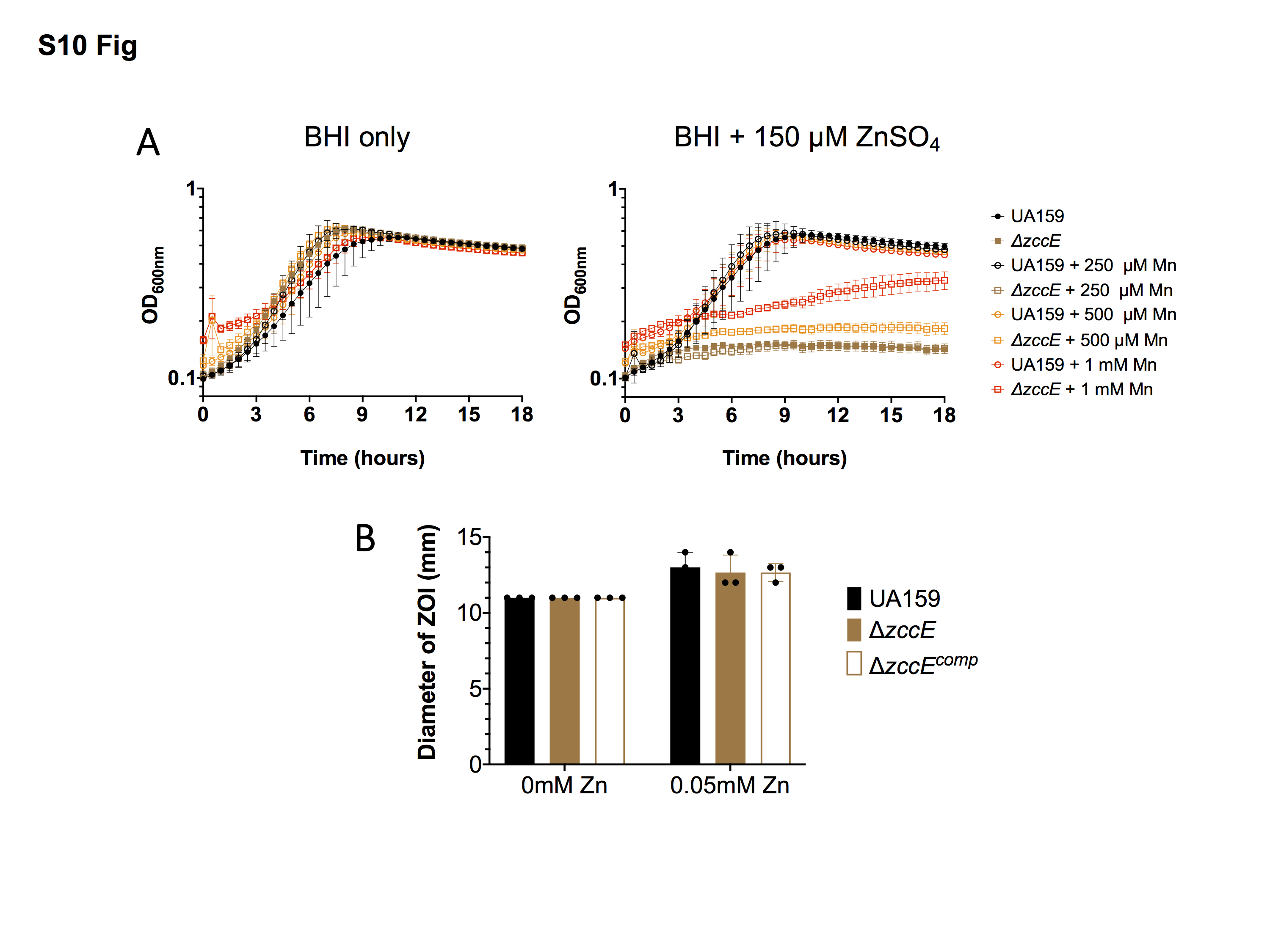

Supplement: S10 Fig — (A) Growth in BHI medium with supplementation of Zn and Mn in different ratios. (B) Growth inhibition zones for S. mutans UA159 and ΔzccE strains grown on BHI agar containing 250 μM Mn with or without Zn supplementation and exposed to filter paper discs saturated with 0.25% H2O2. Data represent means and standard deviations of results from at least 3 independent experiments. (TIF) [file ppat.1010477.s010.tif]
