# Supplementary material for: ZccE is a Novel P-type ATPase That Protects Streptococcus mutans Against Zinc Intoxication
Source: PLoS Pathog. 2022 Aug 8;18(8):e1010477. doi: 10.1371/journal.ppat.1010477 (PMC9387928; doi:10.1371/journal.ppat.1010477)
Supplement: S2 Table — The restriction enzyme sites that were incorporated into the primer sequences are shown in bold. (DOCX) [file ppat.1010477.s012.docx]

**S2 Table. Primers used in the study.**

| **Primer** | **Sequence (5’-3’) *^a^*** | **Application** |
| --- | --- | --- |
| Smu.2057del5’arm1 | GTAAACCAGAAACGAAGG | *zccE* deletion |
| Smu.2057delarm1SphI | GGTTACT**GCATGC**GGCATATTGAAAGCC | *zccE* deletion |
| Smu.2057delarm2SphI | CAGCCTCA**GCATG**CGACTTTTACTAGATTACC | *zccE* deletion |
| Smu.2057del3’arm2 | CTACAGCGCAGCTACATTACC | *zccE* deletion |
| CopYdel5’arm1 | CGGAACAATAGGTTGTTGTGTTGACG | *copYAZ* deletion |
| CopYdel3’ERIarm1 | CCG**GAATTC**GCCCAGACAACACGCATTACTTC | *copYAZ* deletion |
| CopYdelHdIII5’arm2 | CCC**AAGCTT**GGAACCAATTATGAATTGG | *copYAZ* deletion |
| CopYdel3’arm2 | GGTCTGCTGCAGCCTTAACTTCTGG | *copYAZ* deletion |
| Smu2057compBamHI5’ | CGC*G***GATCC**GACATGAAGACCTGAAAACTTAGCC | *zccE* complementation |
| Smu2057compXhoI3’ | CCG**CTCGAG**GCGTTCCTACCAACAATTTTAATGG | *zccE* complementation |
| CopYcompKpn5’ | CGGGGTACCGGAGCTCTCGCCAATCATTTATAGC | *copYAZ* complementation |
| CopYcompXhoI3’ | CCG*CTCGAG*GCCTACTTAAATTTCTGCTCCC | *copYAZ* complementation |
| Smu.2058del5’arm1 | CTGCCAAGGCACAAGGAGAAGC | *zccR* deletion |
| Smu.2058delarm1SphI | ACAT**GCATGC**GCCAATTGTCCAATTGTTAATAACTGTTGCG | *zccR* deletion |
| Smu.2058delarm2SphI | ACAT**GCATGC**CCACATTTAGAAATGCAATACG | *zccR* deletion |
| Smu.2058del3’arm2 | GCTCGGTTTTGGCTTGAATTTGTCACAGG | *zccR* deletion |
| Smu2058compBamHI5 | CGC**GGATCC**GTGTCTTCAACACCCTCCCAG | *zccR* complementation |
| Smu2058compXhoI3 | CCG**CTCGAG**TATGTTTACCCTGTATTATCAACAACG | *zccR* complementation |
| gyrA qRTFwd | CTCCTGACAAGCCGCATAAA | qRT PCR of *gyrA* |
| gyrA qRTRev | GCTCATACGCGCTTCTGTATAA | qRT PCR of *gyrA* |
| zccE qRTFwd | CGCATATATTGATGGCGTTG | qRT PCR of *zccE* |
| zccE qRTRev | TGTCAAACGTCTGGCTTCTG | qRT PCR of *zccE* |
| ZccRover5’Infusion | GGTGGTGGTGCTCGATTACCCTGTATTATCAACAACGT | ZccR purification |
| ZccRover3’Infusion | GACGACAAGGCCATGGTGAAAAAAACGCAACAGTT | ZccR purification |
| EMSA.zccRside | CACAAATAGCTTGACCTTCCCTTTAAGG | EMSA |
| EMSA.zccEside | CATATTGAAAGCCAAGTCATTTTTTGAAGCC | EMSA |
| MntH_EMSA_F | CTTTTCGCAATCTGATTGTTTAG | EMSA |
| MntH_EMSA_R | CATTTTGAAAATCTCTTTTCTAATATAATTG | EMSA |

^a^ Restriction sites are indicated by bold text.
